# Development and temporal validation of an explainable machine-learning model for identifying anxiety symptoms in adults with sudden sensorineural hearing loss

**DOI:** 10.3389/fpubh.2026.2003468

**Published:** 2026-10-23

**Authors:** Miao Yu, Fangyu Yang, Xiaoxia Yu

**Affiliations:** 1 School of Nursing, Capital Medical University, Beijing, China; 2 Beijing Anzhen Hospital, Capital Medical University, Beijing, China

**Keywords:** anxiety symptoms, machine learning, SHAP, sudden sensorineural hearing loss, support vector machine, temporal validation

## Abstract

**Background:**

Sudden sensorineural hearing loss (SSNHL) may be accompanied by substantial psychological distress, yet machine-learning studies have focused mainly on hearing recovery rather than anxiety during acute care.

**Objective:**

To develop, internally validate, temporally evaluate, and interpret machine-learning models for identifying anxiety symptoms in adults hospitalized with first-episode SSNHL after initial treatment and before discharge.

**Methods:**

This single-center retrospective prediction-model study included 512 patients treated at Beijing Anzhen Hospital from February 2022 to August 2023. The 410-patient development cohort was split once, using seed 24, into training (*n* = 287) and internal model-selection (*n* = 123) datasets; 102 later patients formed a same-center temporal-validation cohort. Anxiety symptoms were operationalized as Hamilton Anxiety Rating Scale (HAM-A) score ≥7. Fourteen candidate predictors entered LASSO, Boruta, and recursive feature elimination, and eight classifiers were compared. Robustness analyses included full-pipeline repeated nested cross-validation using all 410 development observations, overlapping-symptom exclusion models, logistic comparators, corrected calibration, a development-only transported threshold, HAM-A ≥ 8 sensitivity, and continuous HAM-A severity. The temporal cohort remained excluded from feature selection, preprocessing, tuning, and algorithm selection.

**Results:**

Overall, 320/512 patients (62.5%) met the HAM-A ≥ 7 criterion. Six predictors were retained in the primary analysis: hearing-loss severity, tinnitus, vertigo, sleep status, number of comorbidities, and treatment efficacy. The selected SVM achieved ROC-AUCs of 0.772 internally and 0.763 temporally. In nested cross-validation, pooled ROC-AUC was 0.747 (95% CI 0.696–0.792), PR-AUC 0.822 (0.768–0.867), and Brier score 0.208 (0.196–0.221); calibration-in-the-large was 0.451 and calibration slope 1.570. XGBoost was selected in 8/10 outer evaluations and RF in 2/10; SVM was not selected. Four predictors—hearing-loss severity, sleep status, number of comorbidities, and treatment efficacy—were retained in all outer evaluations. Using HAM-A ≥ 8 reduced prevalence to 57.0% and temporal SVM ROC-AUC from 0.759 to 0.730.

**Conclusion:**

The SVM selected in the primary workflow showed moderate same-center temporal discrimination, but nested validation demonstrated model-selection instability and imperfect calibration. Together with HAM-A threshold and symptom-overlap sensitivity analyses, these findings support an investigational model-development framework rather than superiority of a specific SVM or an implementation-ready rule. Geographic multicenter validation, a prospectively locked model and threshold, and prospective impact assessment are required.

## 1 Introduction

Sudden sensorineural hearing loss (SSNHL) is an acute otologic condition characterized by rapid and unexpected deterioration in hearing, frequently accompanied by tinnitus and vertigo. Beyond the immediate auditory deficit, abrupt hearing loss can disrupt communication, sleep, social functioning, and emotional well-being, creating a substantial psychosocial burden. A recent qualitative systematic review of adult hearing-related conditions identified anxiety, depression, fear, fatigue, social isolation, and impaired confidence as recurrent domains of health-related quality-of-life loss (1). In patients with unilateral SSNHL, prospective data also suggest that changes in hearing and tinnitus are accompanied by measurable changes in anxiety and depressive symptoms during early follow-up (2). These observations support considering psychological status as a clinically relevant component of SSNHL care rather than focusing solely on audiometric recovery.

Anxiety symptoms are particularly relevant because SSNHL occurs abruptly, often without an immediately identifiable cause, and patients must simultaneously confront uncertainty about hearing recovery, intrusive tinnitus or vertigo, and the possibility of persistent functional impairment. Case–control data have shown substantially higher anxiety and depression scores in adults with sudden unilateral hearing loss than in unaffected controls (3). At the population level, a nationwide cohort study found that patients with idiopathic SSNHL subsequently developed affective disorders, including anxiety and depression, more frequently than matched individuals without SSNHL (4). These findings indicate that psychological morbidity is clinically relevant in SSNHL and support incorporating structured identification of anxiety symptoms alongside routine audiological assessment.

Psychological risk is unlikely to be uniform across all patients. Prior population-based evidence found that the association between SSNHL and anxiety disorder varied by age and was most pronounced among younger adults (5). By contrast, comparatively little SSNHL-specific evidence has characterized differences in psychological burden across sex, educational attainment, income, and other sociodemographic groups. Clinically, anxiety symptoms may also arise within a multidimensional context that includes hearing-loss severity, tinnitus, vertigo, sleep disturbance, comorbidity burden, and response to treatment. A multivariable approach that evaluates these characteristics jointly may therefore be more informative than considering isolated factors, provided that model development and evaluation are conducted with safeguards against overfitting and information leakage.

Prediction modeling in SSNHL has expanded rapidly, but most published models have focused on auditory recovery or treatment prognosis rather than concurrent psychological outcomes. A 2024 systematic review identified seven studies comprising 48 machine-learning models for SSNHL outcomes and reported wide variation in discriminative performance; importantly, all included studies were judged at high risk of bias, raising concerns about real-world applicability and underscoring the need for independent validation and more rigorous model-development practices (6). Recent large-cohort work has continued to develop prognostic tools for hearing recovery using routinely available clinical variables (7), while ensemble artificial-intelligence approaches have likewise been explored for SSNHL prognosis (8). However, these efforts address hearing recovery rather than the separate clinical task of identifying anxiety symptoms during the index SSNHL episode, leaving an important gap in psychologically oriented risk stratification.

In the present study, the target population was adults hospitalized with a first episode of SSNHL, and the intended users were otolaryngology physicians, nurses, and other clinicians involved in inpatient SSNHL care. The intended purpose was diagnostic risk identification of anxiety symptoms during the index hospitalization—after initial SSNHL treatment and before discharge—rather than prediction of future psychiatric disease or replacement of formal psychiatric diagnosis. We therefore aimed to develop and internally validate multivariable machine-learning models based on routinely available demographic and clinical information, select a final model according to a defined hierarchical evaluation rule, and assess its performance in a temporally independent validation cohort. We additionally sought to characterize the contribution of retained predictors using model-interpretation methods, with the goal of supporting targeted structured psychological assessment in patients more likely to exhibit anxiety symptoms.

## 2 Methods

### 2.1 Study design, setting, and data sources

This single-center retrospective observational prediction-model study used existing clinical records of patients treated in the Department of Otorhinolaryngology, Beijing Anzhen Hospital, Capital Medical University, Beijing, China, between February 2022 and August 2023. The study was designed to develop and evaluate multivariable machine-learning models for identifying anxiety symptoms among adult patients with sudden sensorineural hearing loss (SSNHL). Reporting was structured in accordance with the TRIPOD+AI recommendations for studies that develop and evaluate prediction models using regression or machine-learning methods (9).

A temporally separated data structure was used. Patients treated from February 2022 through December 2022 constituted the model-development cohort. This cohort was subsequently divided into a training dataset and a held-out internal model-selection dataset. Patients treated from January 2023 through August 2023 formed a same-center temporal-validation cohort. The later cohort was not used in feature selection, hyperparameter tuning, model fitting, or model selection. Because development and temporal evaluation occurred at the same hospital in non-overlapping calendar periods, this manuscript uses the term temporal validation rather than implying geographic or multicenter external validation.

Within each calendar period, the retrospective analysis included existing clinical records of patients who met the eligibility criteria and had completed the relevant assessments during routine clinical care. Clinical assessment forms and clinical fields were checked at the time of care, and incomplete, ambiguous, or apparently abnormal information was clarified against the patient or source records during routine clinical assessment. A complete screening log covering all potentially eligible patients and reason-specific counts for records not represented in the analytical dataset was unavailable. The present prediction-model analysis used these completed clinical records and involved no additional patient contact or new clinical measurements.

### 2.2 Patients and eligibility criteria

The prespecified inclusion criteria were: (1) a confirmed first episode of sudden sensorineural hearing loss (SSNHL) meeting the 2015 Chinese guideline definition used in the source clinical documentation (sudden, unexplained sensorineural hearing loss developing within 72 h with >20 dB loss at at least two contiguous frequencies); and (2) age ≥18 years. The exclusion criteria were: (1) a physician-documented anxiety disorder, depressive disorder, or other clinically established psychiatric disorder preceding the index SSNHL episode, or a reported first-degree family history of psychiatric illness; (2) severe intellectual or cognitive impairment that precluded valid psychological assessment; and (3) incomplete psychological assessment, incomplete audiological evaluation, or absence of completed required routine clinical assessments in the available records. The psychiatric- and family-history exclusions define a restricted target population and should not be interpreted as prerequisites for valid HAM-A administration in routine SSNHL care.

Pre-existing psychiatric disorders were ascertained before outcome assessment through standardized medical-history taking and review of available electronic medical records. A previous psychiatric disorder was considered present when a diagnosis had been established by a qualified physician before the onset of the index SSNHL episode. Standardized psychiatric diagnostic frameworks such as the Mini-International Neuropsychiatric Interview have demonstrated good reliability and validity for the ascertainment of major psychiatric disorders (10).

A positive family psychiatric history was defined as a reported clinically diagnosed psychiatric disorder in at least one first-degree biological relative, including a parent, sibling, or offspring. Family history was obtained by standardized history taking and corroborated with available collateral information or medical documentation when possible (11). Familial aggregation across major psychiatric disorders is documented (12), but exclusion on this basis was an eligibility restriction for the retrospective analysis rather than a requirement for interpreting current anxiety symptoms. This restriction reduces generalizability because patients with personal or familial psychiatric vulnerability may be among those most relevant to psychological screening in routine practice.

Medication history was reviewed at the index presentation. Psychotropic medication use alone did not constitute an exclusion criterion; exclusion was based on the presence of a pre-existing psychiatric disorder for which the medication had been prescribed. Medication indications were therefore considered together with the medical history rather than inferring psychiatric disease solely from the drug class. Psychotropic medications were classified according to the World Health Organization Anatomical Therapeutic Chemical system, including psycholeptics under N05 and antidepressants under N06A.

All patients were managed within the same departmental SSNHL treatment pathway. The initial treatment course was defined as the continuous period from the first SSNHL-directed treatment to the first planned efficacy reassessment or end of the initial treatment course; actual duration was determined from medication-administration and discharge records rather than assumed to be a fixed number of days. Systemic corticosteroid exposure could be verified by agent, route, daily dose, start/end dates, and duration, and intratympanic steroid use before HAM-A was recorded separately when present. Potentially symptom-modifying medication exposure was also recorded when available, including psycholeptics (ATC N05), antidepressants (ATC N06A), and sedative/hypnotic exposure during the 24 h before HAM-A. These medication fields were not among the 14 candidate predictors used in the primary model. Treatment efficacy was determined before HAM-A administration, fixing the intended use point after initial treatment-response assessment and before discharge.

### 2.3 Study size

The study-size planning calculation for the retrospective prediction-model analysis used the conventional events-per-variable (EPV) heuristic (13): with 14 named candidate predictors, an anticipated anxiety-symptom prevalence of approximately 67.3%, and allowance for approximately 10% potentially invalid or incomplete observations, the target was (10 × 14) / 0.673/0.90 ≈ 231, rounded to 232 records. This calculation is reported for transparency but is not treated as evidence that the machine-learning development sample was formally adequate. Under conventional reference-category indicator coding, the 14 candidate variables represent approximately 25 slope parameters and the final six predictors approximately 10 parameters. Modern prediction-model guidance recommends sample-size assessment based on candidate parameters, outcome proportion, anticipated model performance, shrinkage, and precision rather than a universal EPV rule (14). The 410 development observations were the fixed available sample; consequently, this analysis is best regarded as exploratory model development requiring further independent validation.

The retrospective analysis included 410 eligible patient records from February–December 2022 and 102 eligible patient records from January–August 2023, each with completed routine clinical assessments. All 512 records had complete HAM-A and candidate predictor data; none was subsequently removed from the analytical dataset because of missing values in these fields. The later period therefore contributed 102 complete temporal-validation records. A complete record-screening log covering all patients treated in the two calendar periods was unavailable; the number of potentially eligible patients and reason-specific counts for records not included in the analytical dataset could not be determined. The lower number of included records in the later eight-month period can be described, but its causes cannot be quantified from the available source information.

Patient-record flow through the retrospective analysis is summarized separately for the two calendar periods in Supplementary Figure S1.

### 2.4 Outcome: anxiety symptoms

The target outcome was the presence of anxiety symptoms during the index episode of sudden sensorineural hearing loss (SSNHL). Anxiety symptoms were assessed using the clinician-administered 14-item Hamilton Anxiety Rating Scale (HAM-A), originally developed for standardized assessment of anxiety symptom severity (13). Each item is rated from 0 (not present) to 4 (very severe), yielding a total score ranging from 0 to 56, with higher scores indicating greater anxiety severity. The HAM-A has demonstrated acceptable reliability, validity, and sensitivity to clinical change (15).

For the primary analysis, anxiety status followed the binary outcome definition established for the study dataset: HAM-A total score ≥7 was coded as meeting the study criterion, and <7 as not meeting the criterion. Patient-level data included all 14 HAM-A item scores and the total score for every patient. Item sums matched the recorded total score in all 512 records, and the binary outcome agreed exactly with HAM-A total score ≥7 in 512/512 records. The instrument was the Chinese-language 14-item Hamilton Anxiety Rating Scale (HAM-A/HAMA-14), administered as a clinician-rated scale. The ≥7 boundary is reported as a study-specific operational rule rather than a diagnostic threshold for an anxiety disorder. A widely cited severity-calibration study proposed scores ≤7 as no/minimal anxiety and 8–14 as mild anxiety (16); therefore, sensitivity analyses used a ≥ 8 boundary and continuous HAM-A severity. The ≥7 boundary predated the present *post hoc* robustness analyses.

No independent anxiety questionnaire, structured psychiatric diagnostic interview, or second criterion-standard anxiety measure was collected in the archived study dataset. Therefore, the overlap analyses evaluate robustness of the HAM-A-based construct rather than criterion validity against an independent anxiety measure.

HAM-A assessment was performed during the index hospitalization after completion of the initial SSNHL treatment course and assessment of treatment response, and before discharge. Treatment efficacy and the other candidate predictors had therefore been ascertained before determination of the anxiety outcome. The same assessment window and temporal sequence were used in both the development and temporal-validation cohorts.

The HAM-A was administered face-to-face during routine clinical care after completion of the initial treatment-response assessment and before discharge. Assessors were clinical physicians, clinical psychology/mental-health professionals, or nurses/research staff with patient-assessment experience who had completed HAM-A training and competency assessment. Training covered scale structure and time frame, standardized interviewing, 0–4 item anchors, communication with patients experiencing hearing impairment, and distinction between acute SSNHL/hospital somatic symptoms and anxiety-related somatic manifestations. The same Chinese-language HAMA-14 framework and scoring anchors were used across assessors.

No completed double-rating dataset was available for a cohort-specific inter-rater reliability estimate. Accordingly, no study-specific ICC or κ is reported, and the standardized assessor-training procedures should not be interpreted as evidence that formal inter-rater reliability was quantified in this cohort.

Outcome assessors were not blinded to routine demographic and clinical information because HAM-A assessment occurred within clinical care; they did not have access to feature-selection results, model specifications, predicted probabilities, or other model outputs. Criterion overlap remains important because HAM-A item 4 assesses insomnia, item 8 includes somatic sensory symptoms that can include tinnitus, and item 13 includes autonomic symptoms such as giddiness, whereas sleep status, tinnitus, and vertigo were candidate predictors. These predictors were recorded independently from the corresponding HAM-A item scores. Across all 512 patients, the corresponding predictor-item Spearman correlations were weak (sleep status versus HAM-A insomnia, *ρ* = 0.076; tinnitus versus HAM-A somatic sensory, *ρ* = 0.070; vertigo versus HAM-A autonomic, *ρ* = 0.034). These findings argue against simple duplicate coding but do not eliminate semantic criterion contamination because the overlapping HAM-A items still contribute to the total score. The primary model should therefore be interpreted as identifying the study-specific HAM-A symptom criterion rather than anxiety demonstrably independent of acute sleep or otologic symptom burden.

### 2.5 Candidate predictors and measurement

Candidate predictors were defined before model fitting from demographic, socioeconomic, disease-related, symptom, treatment-response, and comorbidity information available in the study database. Fourteen predictors were entered into the initial feature-selection workflow: sex, age group, education level, monthly income, recent stressful life events, disease duration, laterality of hearing loss, hearing-loss severity, tinnitus, vertigo, aural fullness, sleep status, number of comorbidities, and treatment efficacy. All 14 candidate predictors were entered directly into the three-method feature-selection workflow. No univariable or multivariable logistic regression analysis was performed or used for predictor screening or feature selection.

Sex was categorized as male or female, and age as <40 versus ≥40 years. Age was available only in this dichotomized study coding; continuous age was not retained in the analytic dataset, precluding a continuous-age sensitivity analysis. Education was categorized as primary school/illiterate, junior middle or technical secondary school or below, senior high school or junior college, bachelor’s degree, and master’s degree or above. Monthly income was categorized as no income, <3,000 CNY, 3,000–5,999 CNY, 6,000–9,999 CNY, 10,000–20,000 CNY, and >20,000 CNY. Stressful life events, tinnitus, vertigo, and aural fullness were binary; laterality was unilateral/bilateral; and number of comorbidities was a count.

Verification against the source clinical documentation showed that the disease-duration categories refer to days, not years; the table label ‘years’ was a reporting error and has been corrected to <10 days versus ≥10 days without changing the analytical codes. Disease duration specifically represented the onset-to-treatment interval, defined as the time from the patient’s first perceived sudden hearing loss to initiation of the first SSNHL-directed treatment, and was categorized as <10 days or ≥10 days.

Baseline audiometry was defined as the pre-treatment pure-tone audiogram, with air- and bone-conduction thresholds recorded at 250, 500, 1,000, 2000, 3,000, 4,000, and 8,000 Hz. For standardized baseline hearing-severity description, PTA4 was defined as the arithmetic mean of the index-ear air-conduction thresholds at 500, 1000, 2000, and 4,000 Hz. In unilateral SSNHL, the affected ear was the index ear. For simultaneous bilateral SSNHL, the index ear was defined as the ear with the poorer baseline PTA4, with poorer word-recognition score as the first tie-breaker and the right ear as the final tie-breaker. Baseline hearing severity was therefore based on the pretreatment index-ear audiogram, not on post-treatment hearing. Hearing severity was categorized as mild (20 to <35 dB HL), moderate (35 to <50 dB HL), moderately severe (50 to <65 dB HL), severe (65 to <80 dB HL), profound (80 to <95 dB HL), or total hearing loss (≥95 dB HL). No patient in the 512-record analytic dataset had a severity category below 50 dB HL, which is why Table 1 begins with the moderately severe category.

**Table 1:** Baseline characteristics stratified by the study-specific HAM-A criterion across the training, internal model-selection, and temporal-validation datasets.

Characteristic	Training dataset (*n* = 287)			Internal model-selection dataset (*n* = 123)			Temporal-validation dataset (*n* = 102)		
	Does not meet criterion (*n* = 108)	Meets criterion (*n* = 179)	*p value*	Does not meet criterion (*n* = 46)	Meets criterion (*n* = 77)	*P value*	Does not meet criterion (*n* = 38)	Meets criterion (*n* = 64)	*P* value
Sex			0.591			0.834			0.467
Male	55 (50.9)	97 (54.2)		23 (50.0)	40 (51.9)		23 (60.5)	34 (53.1)	
Female	53 (49.1)	82 (45.8)		23 (50.0)	37 (48.1)		15 (39.5)	30 (46.9)	
Age			0.738			0.653			0.634
<40 years	34 (31.5)	53 (29.6)		12 (26.1)	23 (29.9)		13 (34.2)	19 (29.7)	
≥40 years	74 (68.5)	126 (70.4)		34 (73.9)	54 (70.1)		25 (65.8)	45 (70.3)	
Education level			0.800			0.092			0.263
Primary school or illiterate	2 (1.9)	5 (2.8)		3 (6.5)	0 (0.0)		1 (2.6)	0 (0.0)	
Junior middle/technical secondary school or below	10 (9.3)	21 (11.7)		9 (19.6)	8 (10.4)		5 (13.2)	9 (14.1)	
Senior high school or junior college	53 (49.1)	87 (48.6)		18 (39.1)	39 (50.6)		17 (44.7)	34 (53.1)	
Bachelor’s degree	38 (35.2)	54 (30.2)		13 (28.3)	23 (29.9)		14 (36.8)	15 (23.4)	
Master’s degree or above	5 (4.6)	12 (6.7)		3 (6.5)	7 (9.1)		1 (2.6)	6 (9.4)	
Income level			0.298			0.340			0.643
No income	4 (3.7)	3 (1.7)		2 (4.3)	1 (1.3)		1 (2.6)	2 (3.1)	
<3,000 CNY	3 (2.8)	10 (5.6)		1 (2.2)	1 (1.3)		2 (5.3)	2 (3.1)	
3,000–5,999 CNY	45 (41.7)	76 (42.5)		20 (43.5)	32 (41.6)		19 (50.0)	33 (51.6)	
6,000–9,999 CNY	36 (33.3)	45 (25.1)		11 (23.9)	26 (33.8)		5 (13.2)	12 (18.8)	
10,000–20,000 CNY	19 (17.6)	39 (21.8)		12 (26.1)	13 (16.9)		11 (28.9)	12 (18.8)	
>20,000 CNY	1 (0.9)	6 (3.4)		0 (0.0)	4 (5.2)		0 (0.0)	3 (4.7)	
Stressful life events			0.318			0.502			0.545
No	68 (63.0)	102 (57.0)		32 (69.6)	49 (63.6)		26 (68.4)	40 (62.5)	
Yes	40 (37.0)	77 (43.0)		14 (30.4)	28 (36.4)		12 (31.6)	24 (37.5)	
Disease duration			0.167			0.144			0.003
<10 days	28 (25.9)	34 (19.0)		15 (32.6)	16 (20.8)		15 (39.5)	9 (14.1)	
≥10 days	80 (74.1)	145 (81.0)		31 (67.4)	61 (79.2)		23 (60.5)	55 (85.9)	
Laterality			0.083			0.098			0.314
Unilateral	102 (94.4)	158 (88.3)		43 (93.5)	64 (83.1)		36 (94.7)	56 (87.5)	
Bilateral	6 (5.6)	21 (11.7)		3 (6.5)	13 (16.9)		2 (5.3)	8 (12.5)	
Hearing loss severity			<0.001			0.063			0.119
Moderately severe (50 to <65 dB HL)	28 (25.9)	17 (9.5)		9 (19.6)	5 (6.5)		7 (18.4)	4 (6.2)	
Severe (65 to <80 dB HL)	41 (38.0)	57 (31.8)		21 (45.7)	34 (44.2)		19 (50.0)	27 (42.2)	
Profound (80 to <95 dB HL)	34 (31.5)	81 (45.3)		15 (32.6)	30 (39.0)		10 (26.3)	28 (43.8)	
Total hearing loss (≥95 dB HL)	5 (4.6)	24 (13.4)		1 (2.2)	8 (10.4)		2 (5.3)	5 (7.8)	
Tinnitus			0.068			0.009			0.036
No	34 (31.5)	39 (21.8)		19 (41.3)	15 (19.5)		15 (39.5)	13 (20.3)	
Yes	74 (68.5)	140 (78.2)		27 (58.7)	62 (80.5)		23 (60.5)	51 (79.7)	
Vertigo			0.007			0.055			0.096
No	72 (66.7)	90 (50.3)		32 (69.6)	40 (51.9)		26 (68.4)	33 (51.6)	
Yes	36 (33.3)	89 (49.7)		14 (30.4)	37 (48.1)		12 (31.6)	31 (48.4)	
Aural fullness			0.321			0.983			0.355
No	56 (51.9)	82 (45.8)		21 (45.7)	35 (45.5)		22 (57.9)	31 (48.4)	
Yes	52 (48.1)	97 (54.2)		25 (54.3)	42 (54.5)		16 (42.1)	33 (51.6)	
Sleep status			<0.001			0.003			0.026
Good	49 (45.4)	32 (17.9)		21 (45.7)	16 (20.8)		17 (44.7)	13 (20.3)	
Fair	40 (37.0)	67 (37.4)		16 (34.8)	25 (32.5)		13 (34.2)	27 (42.2)	
Poor	19 (17.6)	80 (44.7)		9 (19.6)	36 (46.8)		8 (21.1)	24 (37.5)	
Number of comorbidities, median (IQR)	1 (0–2)	1 (0–2)	0.124	1 (0–1)	1 (0–2)	0.068	1 (0–2)	1 (0–2)	0.849
Treatment efficacy			<0.001			0.264			<0.001
Markedly effective	52 (48.1)	30 (16.8)		17 (37.0)	18 (23.4)		24 (63.2)	10 (15.6)	
Effective	41 (38.0)	113 (63.1)		24 (52.2)	50 (64.9)		12 (31.6)	44 (68.8)	
Ineffective	15 (13.9)	36 (20.1)		5 (10.9)	9 (11.7)		2 (5.3)	10 (15.6)	

Data are presented as n (%); percentages were calculated within each HAM-A-criterion column. Number of comorbidities is presented as median (IQR). p values compare patients meeting versus not meeting the study-specific HAM-A ≥ 7 criterion within each dataset and are two-sided descriptive comparisons. Pearson’s chi-square test was used when expected cell counts were adequate; Fisher’s exact test for sparse 2 × 2 tables; a fixed-margin Monte Carlo exact procedure (100,000 replicates; seed = 20,260,917) for sparse multi-category tables; and the Mann–Whitney U test for number of comorbidities. Disease duration is in days. CNY, Chinese yuan; dB HL, decibels hearing level; IQR, interquartile range.

Sleep status was collected independently of HAM-A item 4 using a single patient-rated question referring to the seven days before admission: ‘Overall, how would you describe your sleep during the 7 days before this admission?’ Responses were recorded directly as good, fair, or poor and were not derived from HAM-A or PSQI. Treatment efficacy was based on the fixed set of frequencies affected on the baseline audiogram and the mean hearing-threshold improvement at the post-treatment audiogram: the source four-level 2015 Chinese-guideline framework defined cured as recovery to normal, the contralateral-ear level, or the pre-episode level; markedly effective as mean improvement ≥30 dB; effective as 15 to <30 dB; and ineffective as <15 dB. For the three-level model variable, cured and markedly effective were combined as ‘markedly effective’, while the other two levels were retained. The primary post-treatment audiogram was prespecified within 7–14 days after treatment initiation, using the examination closest to day 10 and occurring before HAM-A; if more than one audiogram occurred in the window, the closest to the target day was used rather than the best response. These operational definitions were common to the development and temporal-validation periods.

### 2.6 Data quality, missing data, preprocessing, and dataset partitioning

The development cohort was divided 7:3 by stratified random sampling on the binary HAM-A criterion in R software version 4.4.3 using the reproducible random seed 24, yielding 287 training and 123 internal model-selection observations. This was the only 7:3 split generated; no alternative random seeds or candidate splits were examined. Baseline characteristics were compared descriptively only after the split had been created. The reported *p* > 0.05 checks did not trigger reselection of the seed or repartitioning of the data, and model-performance results were not used to choose the split. Because the 123-patient subset was nevertheless used for between-algorithm model selection, it is described as an internal model-selection dataset rather than an unbiased final test set.

In the primary analysis, each of the 14 candidate predictors entered the feature-selection design matrix as one coded numeric variable rather than being expanded into separate indicator terms. Thus, ordinal or multi-level variables were represented by their study coding in LASSO, Boruta, RFE, and the primary candidate-model workflow. For multicollinearity diagnostics and the *post hoc* logistic comparator analyses, categorical predictors were instead represented by reference-category indicator variables. Centering and scaling were applied to LR, SVM, KNN, and the neural network; tree-based algorithms used the original predictor scale. This difference in encoding is reported explicitly because numeric coding of ordered categories imposes different structural assumptions from indicator coding.

Data completeness and quality were checked during routine clinical care and preparation of the retrospective analytical database. During routine clinical assessment, incomplete or ambiguous responses and clinical fields were checked against the patient or source information and clarified at the time of care. The final analytical dataset comprised 512 eligible patient records with no missing values in the primary outcome, the 14 HAM-A items/total score, or the 14 candidate predictor fields. No record was subsequently removed from this dataset because of unresolved missingness in these variables, and no missing-data imputation was performed. A complete screening/exclusion log for all patients treated during the source periods was unavailable; therefore, the total potentially eligible population and reason-specific counts for records not included in the analytical dataset could not be reconstructed. No additional patient contact was undertaken for the retrospective analysis.

Potentially abnormal or extreme values were reviewed individually during routine clinical documentation and finalization of the retrospective analytical database. Primary source records included original audiograms, electronic medical records, medication-administration records, nursing admission assessments, discharge records, and original HAM-A forms. Suspected recording errors, internally inconsistent responses, or implausible values were checked against the relevant source information and clarified when necessary. Verified clinically plausible values were retained, erroneous entries were corrected before database finalization, and no patient record included in the analytical dataset was removed solely on the basis of an automated statistical outlier rule.

Class imbalance during primary model development was addressed using fold-internal synthetic minority over-sampling (SMOTE). Within each repeated cross-validation resample, SMOTE was applied only to the training portion, while the corresponding validation fold retained its observed class distribution. In the 287-patient training dataset, HAM-A < 7 (‘No’) was the minority class (108/287, 37.6%) and HAM-A ≥ 7 (‘Yes’) was the majority class (179/287, 62.4%); therefore, the ‘No’ class was oversampled. SMOTE was implemented through the sampling function in caret:trainControl using K = min(5, n_min-1), where n_min was the minority-class count in the current training fold, and dup_size = 1, generating one synthetic observation per original minority observation. Applied to the complete training dataset, this setting would increase the minority class from 108 to 216 observations, compared with 179 majority-class observations (No:Yes ≈1.21:1), with fold-specific counts varying across resamples. If a resample contained insufficient minority observations for neighbor-based synthesis or the SMOTE procedure failed, random up-sampling with replacement was used as a fallback. LightGBM followed the same training-fold-only principle. No SMOTE, oversampling, undersampling, or class weighting was applied to the 123-patient internal model-selection dataset or the 102-patient temporal-validation cohort, and feature selection was performed on the original training data before any oversampling.

### 2.7 Feature selection

Feature selection was restricted to the training dataset to avoid information leakage. Three complementary procedures were used: least absolute shrinkage and selection operator (LASSO), Boruta, and recursive feature elimination (RFE). All 14 prespecified candidate predictors entered each feature-selection procedure, and the final predictor set was defined exclusively as the intersection of predictors retained by all three approaches.

For LASSO, binomial logistic regression with an L1 penalty was fitted and the penalty parameter lambda was selected by 10-fold cross-validation using binomial deviance. Both lambda.min, corresponding to the minimum cross-validated deviance, and lambda.1se, the largest lambda within one standard error of the minimum, were displayed. Predictors with non-zero coefficients at lambda.min were carried forward to the three-method intersection. L1 penalization can shrink selected coefficients exactly to zero, thereby combining regularization and variable selection.

Boruta was used as an all-relevant feature-selection method based on random-forest variable importance and comparison with permuted shadow features. The procedure was run with a fixed random seed of 2026 and a maximum of 500 importance-source runs (maxRuns = 500). Variables remaining Tentative after the initial Boruta procedure were resolved using TentativeRoughFix. Only variables ultimately classified as Confirmed were retained (withTentative = FALSE) for the subsequent three-method intersection with LASSO and RFE.

RFE used random forest as the base learner and evaluated predictor subset sizes under repeated 10-fold cross-validation with three repeats, using ROC-AUC as the optimization metric. The subset with the highest mean cross-validated ROC-AUC was selected. RFE iteratively removes less useful variables to obtain a more compact predictive feature set.

After the intersection was determined, multicollinearity among retained predictors was examined using variance inflation factor (VIF), tolerance, and the condition index of the standardized predictor matrix. VIF < 5, tolerance >0.20, and condition index <10 were used as diagnostic reference values for problematic collinearity.

### 2.8 Model development and hyperparameter tuning

Eight binary classifiers were developed using the six-predictor primary feature set: logistic regression (LR), radial-basis-function support vector machine (SVM), gradient boosting machine (GBM), neural network, random forest (RF), extreme gradient boosting (XGBoost), k-nearest neighbors (KNN), and Light Gradient Boosting Machine (LightGBM). The seven caret-based models used repeated stratified 10-fold cross-validation with five repeats, training-fold-only SMOTE, class probabilities, and PR-AUC as the hyperparameter-tuning metric. LR, SVM, KNN, and the neural network were centered and scaled; tree-based models used their native predictor scale. LightGBM used the same repeated fold assignments and training-fold-only SMOTE, with ROC-AUC monitored for early stopping. Between-algorithm selection was based on observed ROC-AUC in the 123-patient internal model-selection subset. The final KNN specification was kmax = 10, distance = 1, and kernel = ‘optimal’.

### 2.9 Model performance evaluation and final-model selection

Candidate-model performance was first summarized in the 123-patient internal model-selection dataset. The model with the highest observed ROC-AUC in that subset was then evaluated in the 102-patient same-center temporal-validation cohort. ROC-AUC was the primary model-selection measure and PR-AUC a complementary discrimination measure; ROC-AUC 95% confidence intervals were calculated with the DeLong method. Because the same 123 observations were used to choose among eight algorithms, performance for the selected winner is not an unbiased internal test estimate. For the primary temporal evaluation, the selected SVM was the model fitted using the 287-patient training dataset and was applied to the 102-patient temporal cohort without refitting on all 410 development patients. The stored 102-case SVM probability file yielded the same temporal ROC-AUC (0.763), PR-AUC (0.833), and Brier score (0.215), confirming consistency of the primary evaluation pathway. A separate *post hoc* sensitivity analysis refit the complete development pipeline on all 410 development observations and applied the locked result once to the 102-patient temporal cohort.

Overall probabilistic prediction error was summarized using the Brier score (17). In the primary analysis, calibration plots used five equal-width probability bins with bin midpoints on the horizontal axis. Because bin midpoints need not equal the mean predicted risk within each bin, the *post hoc* calibration analysis used the actual mean predicted probability in each non-empty bin, reported bin sample sizes and 95% Wilson intervals for observed proportions, and estimated calibration-in-the-large and calibration slope with 95% confidence intervals. Calibration was interpreted separately from discrimination because a similar ROC-AUC across calendar periods does not establish probability calibration (18).

ROC-AUCs of the candidate models in the internal model-selection dataset were compared pairwise using the correlated-ROC DeLong procedure (19). Pairwise comparisons were based on two-sided unadjusted *p* values, and no correction for multiple comparisons was applied. For graphical presentation, the pairwise DeLong results were transformed as −log10(P) and displayed in a heatmap, with significance symbols indicating comparisons with nominal *p* < 0.05. Because these pairwise comparisons were exploratory and no multiplicity adjustment was performed, the corresponding significance indicators were interpreted as unadjusted exploratory comparisons.

For the seven caret-based models, decision thresholds for the 123-patient internal model-selection subset were selected by maximizing F1 on out-of-fold predictions from repeated cross-validation within the 287-patient training dataset and were then applied unchanged to the internal model-selection subset; LightGBM used the analogous training out-of-fold procedure. In the primary temporal analysis, the SVM threshold of 0.216 was selected within the 102-patient temporal-validation dataset and is therefore treated as an exploratory, dataset-specific operating point. For the transported-threshold sensitivity analysis, a threshold was selected using development data only, the model was refit on all 410 development observations, and that fixed threshold was applied unchanged to the temporal cohort.

Decision-curve analysis (DCA) was performed as an exploratory decision-analytic assessment to examine the standardized net benefit of model-guided risk classification across a range of threshold probabilities relative to treat-all and treat-none strategies (20). No specific downstream clinical action or prespecified clinically actionable threshold was defined for a positive model decision. Accordingly, DCA was used to characterize potential decision-analytic value and was not used to determine an implementation threshold or to establish clinical utility.

The primary model-selection hierarchy carried forward the candidate with the highest observed ROC-AUC in the 123-patient internal model-selection subset, which was SVM, with PR-AUC, Brier score, calibration, threshold-dependent metrics, and DCA treated as complementary. This selection is retained as the primary analysis, but the small AUC differences among several candidates and discordant PR-AUC/Brier rankings do not establish clinically important superiority of SVM. Temporal comparator analyses are treated as *post hoc* benchmarks and are not used to select a replacement algorithm after inspection of the 2023 cohort.

Supplementary Figure S2 summarizes the chronology of the primary development/model-selection workflow, the *post hoc* analyses, and the full-pipeline nested cross-validation used as a robustness analysis, while showing that the 102-patient temporal cohort remained excluded from feature selection, preprocessing, tuning, and between-algorithm selection in the nested analysis.

### 2.10 Model interpretation

The SVM selected in the primary workflow was interpreted using SHapley Additive exPlanations (SHAP). The explained dataset comprised all 287 training observations, and the background dataset comprised all 123 internal model-selection observations. SHAP values were calculated on the predicted probability scale for the positive (‘Yes’) class with interactions disabled and seed 2026. Global displays comprised a beeswarm plot and mean absolute SHAP values. Rows 76 and 86 of the training dataset were used as illustrative local examples; they were not selected by a prespecified statistical representativeness rule. Smoothed dependence plots across coded categorical/ordinal predictors were not retained because interpolated intermediate values and algorithmically identified turning points are not clinically interpretable for those variables. SHAP values are treated as model attributions rather than causal effects.

### 2.11 Statistical analysis, software, reporting, and ethics

Baseline characteristics were summarized by whether patients met the study-specific HAM-A ≥ 7 criterion within the training, internal model-selection, and temporal-validation datasets. Categorical variables were reported as *n* (%) and compared using Pearson’s chi-square test when expected cell counts were adequate, Fisher’s exact test for sparse 2 × 2 tables, and a fixed-margin Monte Carlo exact procedure (100,000 replicates; seed 20,260,917) for sparse multi-category tables. Number of comorbidities was reported as median (interquartile range) and compared using the two-sided Mann–Whitney U test. These P values were descriptive and were not interpreted as proof of equivalence or used as predictor-screening criteria.

The primary analyses were performed in R version 4.4.3 using glmnet, Boruta, caret, randomForest, kernlab, gbm, nnet, xgboost, kknn, lightgbm, pROC, PRROC, kernelshap, shapviz (version 0.5.0), and rmda. The full-pipeline nested robustness analysis was run separately in R version 4.4.2 on Windows 11; exact package versions and session information are documented in the accompanying versioned reproducibility materials. *Post hoc* comparator, threshold-transport, HAM-A threshold-sensitivity, continuous-severity, and predictor-selection sensitivity analyses were performed in Python 3.13.5 using scikit-learn 1.8.0 and SciPy 1.17.0. An accompanying versioned reproducibility package contains the analysis code, predictor/data dictionary, software and package-version information, resampling information, and relevant model objects/outputs.

The random seed for the single stratified 7:3 development split was 24, implemented in R software version 4.4.3. No alternative 7:3 seeds or splits were evaluated; baseline *p*-value checks were descriptive assessments performed after the split and did not lead to seed reselection or repartitioning. Seed 2026 was used for LASSO, Boruta, RFE, repeated-cross-validation fold generation, and SHAP computation; model-specific seeds were 2027, 2028, 2029, 2030, 2031, 2032, 2033, and 2034 for LR, SVM, GBM, neural network, RF, XGBoost, KNN, and LightGBM, respectively. The full-pipeline nested analysis used outer-resampling seed 240,925 and recombined all 410 development observations; the temporal cohort was not used in outer-fold construction, feature selection, preprocessing, hyperparameter tuning, winner selection, threshold selection, or probability fitting.

The study was conducted in accordance with the Declaration of Helsinki. The source HAM-A assessments, audiometry, and other clinical variables analyzed in this study were generated exclusively during routine clinical care from February 2022 through August 2023. The subsequent retrospective secondary prediction-model analysis of these existing clinical records was approved by the Ethics Committee of Beijing Anzhen Hospital, Capital Medical University (approval no. KS2024124), and the requirement for informed consent for this retrospective analysis was waived by the Ethics Committee. The analysis used completed records only and involved no additional patient contact or new clinical measurements.

### 2.12 *Post hoc* sensitivity and transport analyses

All analyses in this subsection were conducted after completion of the primary temporal evaluation and were designated *post hoc*; they were not used to redefine the primary model. Patient-level HAM-A data included all 14 item scores and total scores. Data integrity was checked by confirming that the 14 items summed exactly to the recorded total and that the binary outcome equaled HAM-A total score ≥7 for every patient. Total-score distributions, the number scoring exactly 7, and item-level summaries were then reported. For outcome-threshold sensitivity, HAM-A total score ≥8 was defined as the positive class and the six-predictor SVM was refit on all 410 development observations using the same coded predictors and prespecified SVM hyperparameters; the fitted specification was evaluated in the 102-patient temporal cohort without threshold or model selection in that cohort. Predictor-selection sensitivity was also assessed using all 410 development observations under both HAM-A ≥ 7 and ≥8 with identical Python procedures. LASSO used standardized predictors and 10-fold cross-validated L1 logistic regression with binomial log-loss; a Boruta-style all-relevant random-forest procedure used 50 independent shadow-feature runs with multiplicity-adjusted hit testing and a median-importance rough fix for unresolved features; and random-forest recursive feature elimination used 5-fold cross-validated ROC-AUC. The three-method intersection was then compared between the two outcome thresholds. This Python analysis was used only to quantify threshold-related selector stability and did not replace the primary R-based feature-selection analysis.

To assess incremental value against simpler strategies, three development-fitted logistic comparators were evaluated in the temporal cohort without reselecting the winner there: a one-hot-encoded logistic model using the same six predictors; a parsimonious symptom-only logistic model using tinnitus, vertigo, and sleep status; and an L2-penalized logistic model using all 14 archived candidate predictors with penalty tuning restricted to development data. A six-predictor SVM was also evaluated by repeated stratified 10-fold cross-validation with five repeats across the full development cohort as a locked-model robustness analysis. This locked-SVM resampling is reported separately from the full-pipeline nested analysis in Section 2.13, in which feature selection and between-algorithm selection were repeated within the outer resampling structure.

For threshold transport, 10-fold out-of-fold development predictions from the six-predictor SVM were used to select the F1-maximizing threshold separately for the ≥7 and ≥8 outcome definitions; the SVM was then refit to all 410 development observations and each development-derived threshold was applied unchanged to the temporal cohort. Threshold-dependent 95% confidence intervals for the primary ≥7 analysis were obtained by 5,000 stratified bootstrap resamples of the temporal cohort with predictions and threshold fixed. *Post hoc* calibration used actual bin-specific mean predicted probabilities, Wilson confidence intervals for observed proportions, calibration-in-the-large, and calibration slope. The temporal prevalence was reported as the no-skill PR baseline, and the development prevalence transported as a constant probability was used as a Brier-score reference. To avoid dependence on an arbitrary dichotomous boundary, continuous HAM-A severity was also examined with a one-hot-encoded six-predictor ridge regression; the ridge penalty was selected by 10-fold development-only cross-validation, and temporal performance was summarized using RMSE, MAE, R^2^, Pearson correlation, and Spearman correlation. These comparator, threshold, outcome-sensitivity, continuous-severity, and predictor-selection sensitivity analyses were implemented in Python 3.13.5 with scikit-learn 1.8.0 and SciPy 1.17.0. The full-pipeline nested analysis described below was implemented independently in R and is reported separately.

For the primary SVM, positive-class probabilities were generated directly by the probability-enabled radial-basis-function SVM during model fitting; no separate post-fitting calibration model was used to transform decision scores into probabilities. Centering and scaling, SVM fitting, and probability estimation were performed using development data only. The *post hoc* all-410 SVM sensitivity analyses used the same development-only principle, with scaling and probability estimation fitted before application to the temporal cohort. The primary SVM predictions and performance estimates were generated using the same fold-internal SMOTE training procedure described above.

### 2.13 Full-pipeline nested cross-validation

To evaluate the complete development procedure independently of the single 287/123 split, all 410 development observations were recombined and analyzed using repeated stratified 5-fold outer cross-validation with two repeats (10 outer evaluations). The 102-patient temporal cohort was kept completely locked. Within each outer-training fold, all 14 candidate predictors re-entered LASSO, Boruta, and random-forest RFE. LASSO used 10-fold internal cross-validation; Boruta used maxRuns = 500 and TentativeRoughFix only when Tentative attributes were present; RFE was repeated within the outer-training data. The three-way selector intersection defined the fold-specific feature set. A prespecified two-of-three fallback was available only if the three-way intersection was empty; this fallback was not required in any of the 10 outer evaluations.

The eight candidate algorithms were then tuned inside each outer-training fold using repeated stratified 5-fold inner cross-validation with two repeats. SMOTE was restricted to the inner-training portion of each resample. Hyperparameters were optimized by PR-AUC, after which the candidate algorithm with the highest inner-CV ROC-AUC was selected according to the primary model-selection hierarchy, refitted on the complete outer-training fold, and evaluated once in the untouched outer-test fold. Each patient entered an outer-test fold once per repeat; the two outer-test probabilities were averaged at the patient level before pooled evaluation. ROC-AUC, PR-AUC, Brier score, calibration-in-the-large, and calibration slope were summarized with 2,000 patient-level bootstrap resamples. Feature-selection frequencies and winner-model frequencies were summarized across all 10 outer evaluations.

As a separate development-only sensitivity analysis, the complete selector-plus-eight-algorithm pipeline was rerun once using all 410 development observations. The selected algorithm, its preprocessing, and its development-derived F1 threshold were then locked and applied once to the 102-patient temporal cohort without temporal retuning. This analysis provides an explicit all-410 refit pathway but does not alter the model selected in the primary workflow.

## 3 Results

### 3.1 Patient composition and cohort characteristics

A total of 512 patients meeting the eligibility criteria were included in the retrospective analysis, with complete clinical records for the study variables. The development cohort comprised 410 patients, of whom 256 (62.4%) met the study-specific HAM-A ≥ 7 criterion. The 7:3 split yielded 287 training observations (179 meeting the criterion, 62.4%) and 123 internal model-selection observations (77 meeting the criterion, 62.6%). The same-center temporal-validation cohort comprised 102 patients, including 64 (62.7%) meeting the criterion. Across all records, 320/512 (62.5%) met the ≥7 criterion. All 512 analytical records had complete HAM-A and candidate predictor data, and no record was subsequently removed from this dataset because of missing values in these fields. A complete screening denominator and reason-specific counts for records not included in the analytical dataset were unavailable in the source materials.

Post-split characteristics are summarized in Supplementary Table S1. All training-versus-internal model-selection *p* values exceeded 0.05, but these descriptive tests are not evidence of equivalence. Only one stratified 7:3 split was generated, using seed 24; the *p* values were calculated after that split had been fixed and did not influence seed selection or repartitioning. Within the training data, the HAM-A criterion differed descriptively by hearing-loss severity (*p* < 0.001), vertigo (*p* = 0.007), sleep status (*p* < 0.001), and treatment efficacy (*p* < 0.001). In the internal model-selection subset, tinnitus (*p* = 0.009) and sleep status (*p* = 0.003) differed between criterion groups. In temporal validation, disease duration (*p* = 0.003), tinnitus (*p* = 0.036), sleep status (*p* = 0.026), and treatment efficacy (*p* < 0.001) differed between criterion groups (Table 1). Other descriptive comparisons are shown in Table 1.

HAM-A total-score distributions were: development cohort, mean 10.00 ± 6.08, median 10 (IQR 4.25–15), range 0–28; training dataset, 10.01 ± 6.07, median 10 (IQR 5–15), range 0–28; internal model-selection dataset, 9.97 ± 6.12, median 10 (IQR 4–15), range 0–23; and temporal-validation cohort, 9.73 ± 5.72, median 9.5 (IQR 4–14), range 1–27. Exactly 23/410 development patients scored 7 (17/287 training and 6/123 internal model selection), and 5/102 temporal patients scored 7; overall, 28/512 (5.5%) scored exactly 7.

Item-level HAM-A information was available for all 512 patients. Mean±SD scores and the proportion with an item score >0 were: anxious mood 1.12 ± 1.12 (63.9%); tension 0.97 ± 1.02 (61.3%); fears 0.96 ± 1.00 (59.8%); insomnia 0.96 ± 1.01 (58.6%); intellectual symptoms 0.66 ± 0.83 (47.7%); depressed mood 0.79 ± 0.89 (53.9%); somatic-muscular 0.68 ± 0.85 (47.3%); somatic-sensory 0.61 ± 0.79 (44.7%); cardiovascular 0.51 ± 0.75 (38.3%); respiratory 0.50 ± 0.76 (36.1%); gastrointestinal 0.50 ± 0.71 (39.5%); genitourinary 0.40 ± 0.66 (30.9%); autonomic 0.56 ± 0.78 (41.4%); and behavior at interview 0.74 ± 0.87 (50.8%).

Patient-record flow through the retrospective analysis was as follows: February–December 2022, 410 eligible complete records included → 287 training + 123 internal model-selection records; January–August 2023, 102 eligible complete records included → 102 temporal-validation records. Records subsequently removed from these analytical cohorts because of unresolved HAM-A or candidate predictor missingness: 0. A complete screening denominator and reason-specific counts for records not included were unavailable. This flow is displayed graphically in Supplementary Figure S1.

### 3.2 Feature selection and multicollinearity

LASSO cross-validation identified lambda.mi*n* = 0.0144 and lambda.1se = 0.064 (Figure 1A). At lambda.1se, four predictors retained non-zero coefficients: sleep status, treatment efficacy, hearing-loss severity, and vertigo. At lambda.min, 11 predictors retained non-zero coefficients: sleep status, treatment efficacy, vertigo, tinnitus, hearing-loss severity, laterality, income level, disease duration, aural fullness, number of comorbidities, and sex (Figure 1B; Supplementary Figure S3).

**Figure 1:**
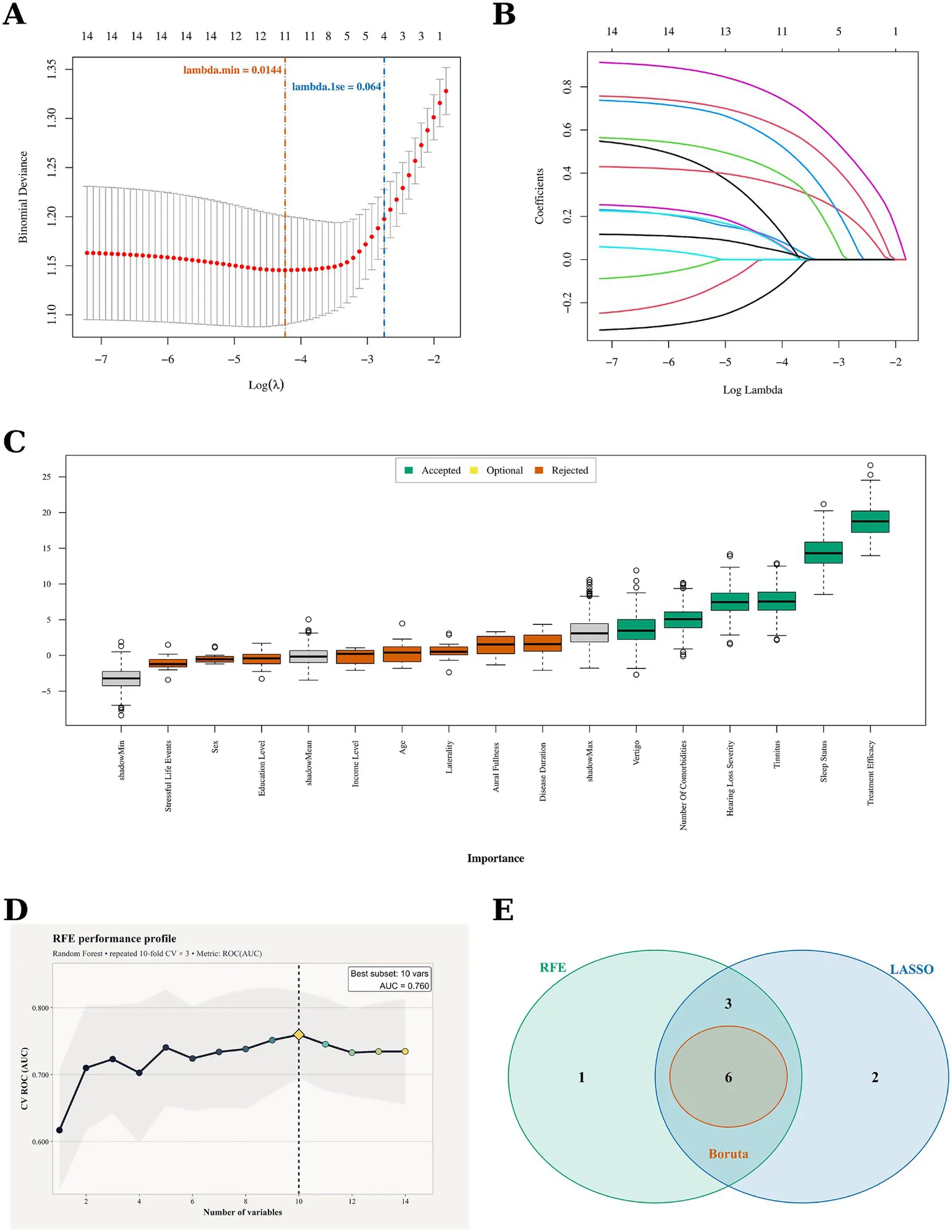
Feature selection and consensus identification using LASSO, Boruta, and recursive feature elimination. **(A)** Ten-fold cross-validation curve for the LASSO model, showing binomial deviance across log(λ); the vertical reference lines indicate λ.min = 0.0144 and λ.1se = 0.064. **(B)** LASSO coefficient profiles showing the trajectories of regression coefficients as a function of log(λ). **(C)** Boruta variable-importance distributions, with variables classified as Accepted, Optional, or Rejected relative to the shadow features. **(D)** Recursive feature elimination (RFE) performance profile based on a random forest with repeated 10-fold cross-validation (3 repeats) and ROC-AUC as the optimization metric; the best subset contained 10 variables with an AUC of 0.760. **(E)** Venn diagram showing the overlap among variables retained by RFE, LASSO, and Boruta; six variables were common to all three methods. LASSO, least absolute shrinkage and selection operator; RFE, recursive feature elimination; CV, cross-validation; ROC, receiver operating characteristic; AUC, area under the curve.

Boruta retained six accepted predictors, whereas RFE achieved its highest repeated-cross-validation ROC-AUC with a 10-predictor subset (mean ROC-AUC = 0.760; Figures 1C,D). The intersection of LASSO, Boruta, and RFE comprised six predictors: hearing-loss severity, tinnitus, vertigo, sleep status, number of comorbidities, and treatment efficacy (Figure 1E). These six variables were used in all subsequent candidate models.

Multicollinearity diagnostics showed no evidence of problematic collinearity among the retained predictors. VIF values ranged from 1.032 to 2.352, all tolerance values were ≥0.425, and the maximum condition index was 3.04 (Supplementary Table S2).

### 3.3 Model training and held-out internal model selection

The model-specific hyperparameters from the primary training workflow are listed in Supplementary Table S3. The selected settings were: SVM, sigma = 0.1 and C = 0.1; GBM, n.trees = 100, interaction.depth = 3, shrinkage = 0.1, and n.minobsinnode = 5; neural network, size = 5, decay = 0.6, and maximum iterations = 500; RF, mtry = 2; XGBoost, nrounds = 200, max_depth = 5, eta = 0.01, gamma = 0.5, colsample_bytree = 0.5, min_child_weight = 1, and subsample = 0.6; KNN, kmax = 10, distance = 1, and kernel = ‘optimal’; and LightGBM, final nrounds = 16 with learning_rate = 0.05, num_leaves = 31, feature_fractio*n* = 0.8, bagging_fractio*n* = 0.8, bagging_freq = 1, and min_data_in_leaf = 10.

In the 123-patient internal model-selection subset, SVM had the highest observed ROC-AUC of 0.772 (95% CI 0.685–0.859), followed closely by XGBoost at 0.767 (95% CI 0.680–0.854) and LR at 0.762 (95% CI 0.675–0.850); the remaining ROC-AUCs were 0.757 for GBM, 0.737 for the neural network, 0.720 for RF, 0.716 for LightGBM, and 0.616 for KNN (Figure 2A). Because this subset was used to choose among the eight algorithms, these values are model-selection performance summaries rather than an unbiased internal test estimate for the selected SVM. Pairwise DeLong comparisons are shown in Supplementary Figure S4A and remain unadjusted exploratory comparisons.

**Figure 2:**
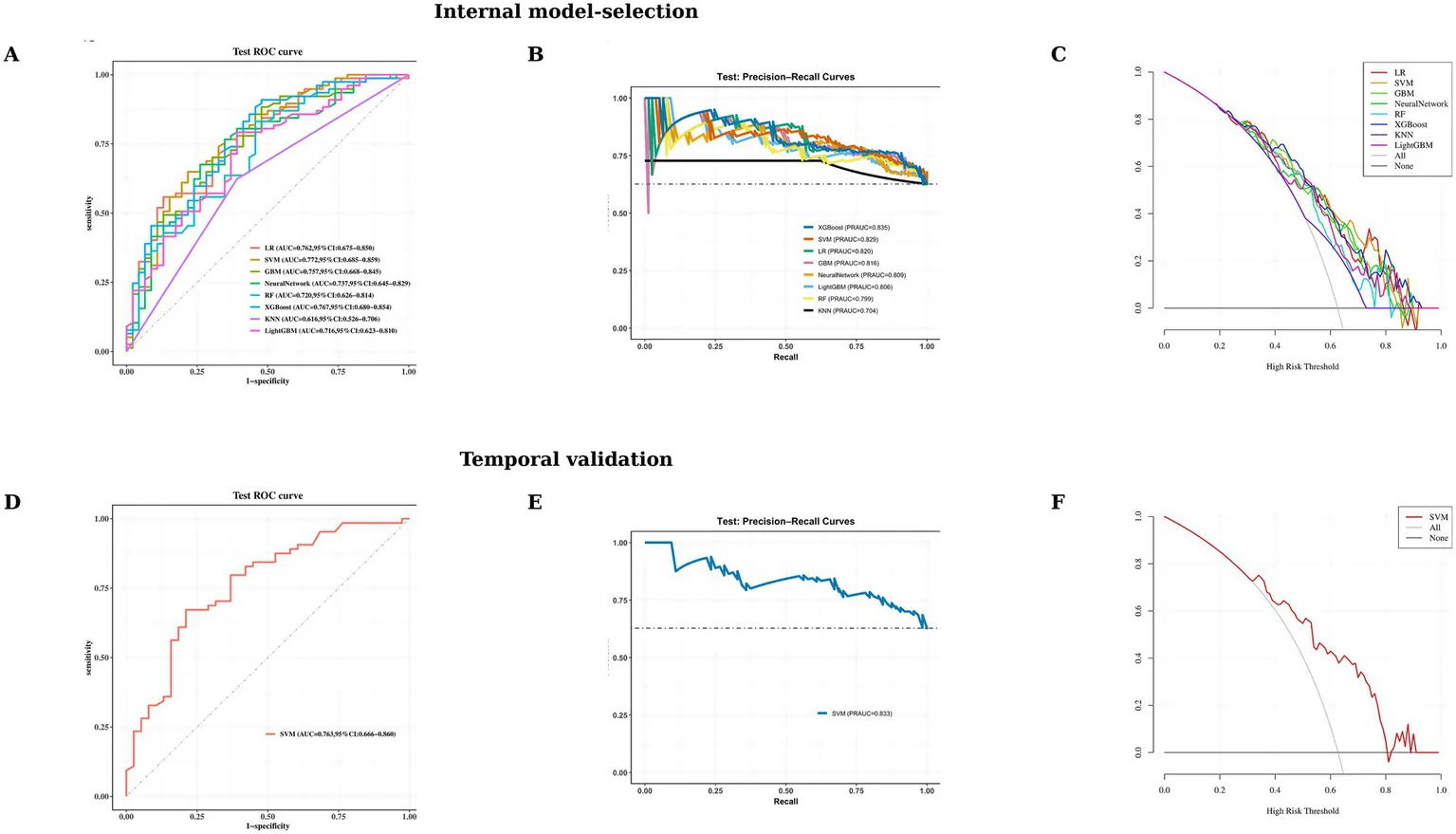
Discrimination and exploratory decision-curve analysis in the internal model-selection and temporal-validation datasets. **(A–C)** Internal model-selection performance of the eight candidate models: **(A)** receiver operating characteristic (ROC) curves with area under the curve (AUC) and 95% confidence intervals; **(B)** precision-recall (PR) curves with PR-AUC values; and **(C)** exploratory decision-curve analysis (DCA). **(D–F)** Temporal validation of the primary support vector machine (SVM): **(D)** ROC curve (AUC = 0.763, 95% CI 0.666–0.860); **(E)** PR curve (PR-AUC = 0.833); and **(F)** exploratory DCA. Temporal calibration is reported separately using actual mean predicted probabilities, bin sample sizes, 95% Wilson intervals, calibration-in-the-large, and calibration slope in Section 3.5 and Table 3. DCA is descriptive because no downstream action and no clinical threshold were prespecified.

PR-AUC values in the internal model-selection dataset were 0.835 for XGBoost, 0.829 for SVM, 0.820 for LR, 0.816 for GBM, 0.809 for the neural network, 0.806 for LightGBM, 0.799 for RF, and 0.704 for KNN (Figure 2B). Brier scores were 0.198 for XGBoost, 0.204 for GBM, 0.210 for SVM, 0.215 for LR, 0.220 for LightGBM, 0.221 for the neural network, 0.227 for RF, and 0.382 for KNN. Exploratory decision curves for all eight candidate models are shown in Figure 2C. Calibration was reassessed separately using actual mean predicted probabilities rather than equal-width-bin midpoints.

The SVM threshold of 0.196 was selected from out-of-fold predictions within the 287-patient training dataset and then transported unchanged to the 123-patient internal model-selection subset. At this training-derived operating point, the reported internal model-selection accuracy was 0.724, sensitivity 0.883, specificity 0.457, precision 0.731, F1 score 0.800, and MCC 0.383 (Table 2), with 68 true positives, 9 false negatives, 21 true negatives, and 25 false positives (Supplementary Figure S5J). These metrics evaluate a training-derived threshold in the model-selection subset, although that subset was also used for between-algorithm selection and is therefore not an unbiased final test set.

**Table 2:** Threshold-dependent performance in the primary internal model-selection and temporal-validation analyses.

Model	Threshold	Accuracy	Sensitivity	Specificity	Precision	F1	MCC
Internal model-selection dataset							
RF	0.210	0.715	0.922	0.370	0.710	0.802	0.362
XGBoost	0.337	0.715	0.922	0.370	0.710	0.802	0.362
LightGBM	0.436	0.675	0.714	0.609	0.753	0.733	0.318
GBM	0.281	0.748	0.883	0.522	0.756	0.814	0.442
Neural network	0.173	0.667	0.909	0.261	0.673	0.773	0.228
SVM	0.196	0.724	0.883	0.457	0.731	0.800	0.383
KNN	0.000	0.626	1.000	0.000	0.626	0.770	0.000
LR	0.186	0.732	0.948	0.370	0.716	0.816	0.408
Temporal-validation dataset							
SVM	0.216	0.735	0.844	0.553	0.761	0.800	0.417

For the internal model-selection rows, thresholds were selected from training-set out-of-fold predictions by maximizing F1 and were then applied unchanged to the 123-patient subset. The temporal SVM threshold of 0.216 was instead optimized within the temporal-validation dataset and is retained only as an exploratory best-observed operating point. The development-only threshold transported unchanged to temporal validation is reported in Section 3.5.

LR, logistic regression; SVM, support vector machine; GBM, gradient boosting machine; RF, random forest; XGBoost, extreme gradient boosting; KNN, k-nearest neighbors; LightGBM, Light Gradient Boosting Machine; F1, F1 score; MCC, Matthews correlation coefficient.

Under the prespecified primary hierarchy, SVM was carried forward because it had the highest observed ROC-AUC in the internal model-selection subset. XGBoost nevertheless had a slightly higher PR-AUC (0.835 versus 0.829) and lower Brier score (0.198 versus 0.210). Accordingly, SVM should not be described as uniformly or clinically superior; simpler comparator models are evaluated *post hoc* without using the temporal cohort to select a replacement winner.

### 3.4 Temporal validation of the primary SVM model

When the six-predictor SVM selected in the primary workflow was applied to the January–August 2023 same-center temporal cohort, ROC-AUC was 0.763 (95% CI 0.666–0.860), PR-AUC was 0.833, and Brier score was 0.215 (Figures 2D,E). The temporal outcome prevalence, 64/102 = 0.627, is the relevant no-skill precision-recall baseline. A constant prediction equal to the development prevalence (256/410 = 0.624) gives a temporal Brier score of approximately 0.234, so the SVM Brier score represents only a modest improvement over that prevalence benchmark. Exploratory temporal DCA is shown in Figure 2F. Corrected calibration using actual mean predicted probabilities is reported in Section 3.5 and Table 3.

**Table 3:** Corrected post hoc temporal calibration using actual mean predicted probabilities.

Predicted-risk bin	*n*	Actual mean predicted risk	Observed proportion (95% CI)
0.0- < 0.2	9	0.103	0.111 (0.020–0.435)
0.2- < 0.4	23	0.266	0.435 (0.256–0.632)
0.4- < 0.6	15	0.528	0.600 (0.357–0.802)
0.6- < 0.8	31	0.732	0.742 (0.568–0.863)
0.8–1.0	24	0.834	0.875 (0.690–0.957)

Using the temporal dataset-specific F1-optimal threshold of 0.216, accuracy was 0.735, sensitivity 0.844, specificity 0.553, precision 0.761, F1 score 0.800, and MCC 0.417 (Table 2), with 54 true positives, 10 false negatives, 21 true negatives, and 17 false positives. These values are explicitly exploratory because the threshold was optimized in the temporal cohort. The threshold-transport analysis instead uses a development-only threshold and is reported in Section 3.5.

### 3.5 *Post hoc* sensitivity, comparator, calibration, and threshold analyses

All analyses in this subsection were conducted after completion of the primary temporal evaluation and are therefore *post hoc*. Using all 410 development observations to refit a standardized six-predictor RBF SVM and evaluating the locked specification in the 102-patient temporal cohort yielded ROC-AUC 0.759, PR-AUC 0.842, and Brier score 0.192. Excluding sleep status gave ROC-AUC 0.754, PR-AUC 0.831, and Brier score 0.189; excluding all three predictors with direct/partial HAM-A symptom overlap (sleep status, tinnitus, and vertigo) gave ROC-AUC 0.743, PR-AUC 0.805, and Brier score 0.198. Thus, overlap does not account for all discrimination, but the reduction after removing overlapping symptoms supports interpreting part of the primary model signal as identification of the acute HAM-A symptom construct rather than anxiety fully independent of sleep/otologic symptoms.

Simple comparator models did not show a clear incremental advantage for SVM. A one-hot six-predictor logistic model yielded temporal ROC-AUC 0.769, PR-AUC 0.840, and Brier score 0.182; a symptom-only logistic model using tinnitus, vertigo, and sleep status yielded 0.690, 0.769, and 0.212, respectively; and an L2-penalized logistic benchmark using all 14 candidate predictors yielded 0.778, 0.848, and 0.178. These *post hoc* results do not justify selecting a new winner on the basis of temporal-cohort performance, but they show that the SVM selected in the primary workflow did not demonstrate clinically important incremental performance over simpler regression benchmarks.

For the six-predictor *post hoc* SVM, calibration-in-the-large was 0.357 (95% CI -0.116 to 0.829) and the calibration slope was 0.841 (95% CI 0.455 to 1.226). Corrected equal-width-bin calibration used actual mean predicted probabilities. The five bins contained *n* = 9, 23, 15, 31, and 24 patients; mean predicted risks were 0.103, 0.266, 0.528, 0.732, and 0.834; observed proportions meeting the HAM-A criterion were 0.111 (95% CI 0.020–0.435), 0.435 (0.256–0.632), 0.600 (0.357–0.802), 0.742 (0.568–0.863), and 0.875 (0.690–0.957), respectively. The confidence intervals are wide, especially in smaller bins, and the 102-patient cohort does not support highly flexible calibration modeling.

The development-only out-of-fold F1-optimal threshold was 0.285. After refitting the six-predictor SVM on all 410 development observations, applying 0.285 unchanged to the temporal cohort classified 78/102 patients for further assessment and left 24 unselected. The confusion matrix was 57 true positives, 21 false positives, 17 true negatives, and 7 false negatives. Accuracy was 0.725 (95% bootstrap CI 0.647–0.804), sensitivity 0.891 (0.813–0.953), specificity 0.447 (0.289–0.605), precision 0.731 (0.675–0.795), negative predictive value 0.708 (0.542–0.870), F1 0.803 (0.746–0.855), and MCC 0.385 (0.189–0.571). Assessing every temporal patient would have an F1 of 0.771, require 102 assessments, and miss no criterion-positive cases; the transported model threshold would spare 24 assessments but miss 7/64 criterion-positive patients. This workload-versus-missed-case tradeoff cannot establish clinical benefit without comparison against usual clinical judgment or a brief direct anxiety screen, neither of which was collected in the archived study data.

As a separate locked-model robustness analysis, repeated stratified 10-fold cross-validation with five repeats across the complete 410-patient development cohort using the fixed six-predictor SVM produced mean ROC-AUC 0.770 (SD 0.075), mean PR-AUC 0.848 (SD 0.058), and mean Brier score 0.190 (SD 0.031). This analysis evaluates the fixed SVM specification only; the full-pipeline nested evaluation of the complete selector-plus-eight-algorithm development procedure is reported separately in Section 3.6.

Changing the operational outcome from HAM-A ≥ 7 to ≥8 reclassified 23/410 development patients (5.6% of the development cohort; 9.0% of those positive at ≥7) and 5/102 temporal patients (4.9%; 7.8% of those positive at ≥7). Prevalence therefore changed from 62.4 to 56.8% in development, from 62.7 to 57.8% in temporal validation, and from 62.5 to 57.0% overall. With the same six predictors and the development-only *post hoc* SVM fitting procedure, temporal ROC-AUC decreased from 0.759 to 0.730, PR-AUC from 0.842 to 0.786, and Brier score increased from 0.192 to 0.209. Under ≥8, calibration-in-the-large was 0.237 (95% CI -0.203 to 0.676) and the calibration slope was 0.873 (95% CI 0.425–1.321), compared with 0.357 and 0.841, respectively, under ≥7 (Table 4).

**Table 4:** *Post hoc* temporal-validation sensitivity and comparator analyses (HAM-A ≥ 7 unless otherwise specified).

*Post hoc* model	ROC-AUC	PR-AUC	Brier	Cal.-in-large	Cal. slope (95% CI)
SVM, six predictors (HAM-A ≥ 7)	0.759	0.842	0.192	0.357	0.841 (0.455–1.226)
SVM, excluding sleep	0.754	0.831	0.189	0.191	1.204 (0.626–1.782)
SVM, excluding sleep/tinnitus/vertigo	0.743	0.805	0.198	0.170	1.412 (0.698–2.126)
Logistic, same six predictors	0.769	0.840	0.182	0.156	0.887 (0.491–1.283)
Logistic, symptoms only	0.690	0.769	0.212	0.093	0.697 (0.260–1.134)
L2 logistic, all 14 predictors	0.778	0.848	0.178	0.154	0.865 (0.485–1.245)
SVM, six predictors (HAM-A ≥ 8 sensitivity)	0.730	0.786	0.209	0.237	0.873 (0.425–1.321)

The *post hoc* Python predictor-selection sensitivity analysis showed component-level changes but a stable final three-method intersection (Supplementary Table S4). Under HAM-A ≥ 7, LASSO retained 12 predictors, the Boruta-style procedure retained four, and RF-RFE selected a 12-predictor subset with mean 5-fold ROC-AUC 0.756. Under HAM-A ≥ 8, LASSO retained 10 predictors, the Boruta-style procedure retained the same four, and RF-RFE selected a 9-predictor subset with mean 5-fold ROC-AUC 0.737. Nine LASSO predictors were common to both thresholds; age, stressful life events, and laterality were selected only at ≥7, whereas education level was selected only at ≥8. For RF-RFE, nine predictors were common to both thresholds, while sex, stressful life events, and aural fullness were retained only at ≥7. Most importantly, the three-method intersection was identical under both definitions: hearing-loss severity, sleep status, number of comorbidities, and treatment efficacy. Thus, moving the HAM-A threshold by one point changed membership within LASSO and RFE but did not alter the final intersection in this *post hoc* selector-stability analysis.

At the ≥8 definition, the development-only 10-fold out-of-fold F1-optimal SVM threshold was 0.330. Applied unchanged after refitting on all 410 development patients, this threshold produced 50 true positives, 9 false negatives, 18 true negatives, and 25 false positives in temporal validation: accuracy 0.667, sensitivity 0.847, specificity 0.419, precision 0.667, negative predictive value 0.667, F1 0.746, and MCC 0.298. In the separate continuous-severity analysis, the development-fitted six-predictor ridge model (10-fold development-selected penalty α = 7.565) achieved temporal RMSE 5.37, MAE 4.32, R^2^ 0.112, Pearson r = 0.337 (*p* < 0.001), and Spearman *ρ* = 0.343 (*p* < 0.001); by comparison, a constant prediction equal to the development mean HAM-A score had RMSE 5.70. This supports a modest continuous symptom-severity signal but does not indicate precise individual severity prediction.

### 3.6 Full-pipeline nested cross-validation

The full-pipeline nested analysis completed all 10 outer evaluations without requiring the prespecified two-of-three feature-selection fallback. After averaging the two outer-test probabilities for each of the 410 development patients, pooled ROC-AUC was 0.747 (95% bootstrap CI 0.696–0.792), PR-AUC 0.822 (95% CI 0.768–0.867), and Brier score 0.208 (95% CI 0.196–0.221). Calibration-in-the-large was 0.451 (95% CI 0.250–0.655) and the calibration slope was 1.570 (95% CI 1.135–2.117), indicating that the nested predictions remained imperfectly calibrated despite moderate discrimination (Table 5). Across individual outer evaluations, mean ROC-AUC was 0.762 (SD 0.062), mean PR-AUC 0.849 (SD 0.038), and mean Brier score 0.215 (SD 0.026).

**Table 5:** Patient-level pooled performance from full-pipeline nested cross-validation.

Metric	Estimate	95% bootstrap CI
ROC-AUC	0.747	0.696–0.792
PR-AUC	0.822	0.768–0.867
Brier score	0.208	0.196–0.221
Calibration-in-the-large	0.451	0.250–0.655
Calibration slope	1.570	1.135–2.117

The development cohort (*n* = 410) was evaluated using repeated stratified 5-fold outer cross-validation with two repeats. Feature selection, preprocessing, inner tuning, and algorithm selection were repeated inside each outer-training fold. Each patient had two outer-test predictions, which were averaged before pooled evaluation. Confidence intervals are percentile intervals from 2,000 patient-level bootstrap resamples. The prespecified two-of-three selector fallback was not required in any outer evaluation.

Feature stability was strongest for hearing-loss severity, sleep status, number of comorbidities, and treatment efficacy, each of which entered the final three-selector intersection in all 10 outer evaluations. Vertigo entered the intersection in 9/10 evaluations and tinnitus in 8/10; income level entered in 3/10 and disease duration in 1/10. The remaining six candidate predictors never entered the final three-way intersection. Algorithm selection was also not invariant: XGBoost was selected in 8/10 outer evaluations and RF in 2/10, whereas the SVM selected in the primary workflow was not the winner in any outer evaluation (Table 6). These findings show that the core predictor signal was more stable than the identity of a single preferred algorithm.

**Table 6:** Feature-selection stability across the 10 outer evaluations of the full-pipeline nested analysis.

Candidate predictor	LASSO (%)	Boruta (%)	RFE (%)	Final intersection (%)
Hearing-loss severity	100	100	100	100
Number of comorbidities	100	100	100	100
Sleep status	100	100	100	100
Treatment efficacy	100	100	100	100
Vertigo	100	100	90	90
Tinnitus	100	90	90	80
Income level	100	30	100	30
Disease duration	90	10	50	10
Age	40	0	40	0
Aural fullness	30	0	60	0
Education level	40	0	90	0
Laterality	80	0	20	0
Sex	50	0	50	0
Stressful life events	40	0	40	0

Percentages denote the proportion of the 10 outer evaluations in which each predictor was retained by the stated selector or by the final three-way intersection. XGBoost was selected as the winning algorithm in 8/10 outer evaluations (80%) and RF in 2/10 (20%); no other algorithm was selected as the outer-fold winner.

In the separate all-410 locked sensitivity refit, the three-selector intersection contained seven predictors: income level, hearing-loss severity, tinnitus, vertigo, sleep status, number of comorbidities, and treatment efficacy. RF had the highest development-only inner-CV ROC-AUC (0.7853), narrowly above XGBoost (0.7852), and was therefore selected according to the prespecified hierarchy. The development-derived F1 threshold was 0.432. Applied once, unchanged, to the 102-patient temporal cohort, this locked RF yielded ROC-AUC 0.762, PR-AUC 0.788, Brier score 0.194, calibration-in-the-large 0.453, and calibration slope 0.678. At threshold 0.432, accuracy was 0.716, sensitivity 0.828, specificity 0.526, precision 0.746, and F1 0.785, with 53 true positives, 11 false negatives, 20 true negatives, and 18 false positives. Because this analysis was conducted after completion of the primary temporal evaluation, it is treated as a *post hoc* sensitivity analysis and not as a replacement model-selection result.

### 3.7 SHAP interpretation of the primary SVM model

SHAP analysis ranked sleep status as the most influential predictor by mean absolute SHAP magnitude, followed by tinnitus and treatment efficacy, with hearing-loss severity, vertigo, and number of comorbidities contributing smaller overall magnitudes (Figures 3A,B). The beeswarm distribution illustrated substantial heterogeneity in the direction and magnitude of feature contributions across individual patients. Smoothed dependence plots were not retained because interpolated intermediate values and turning-point annotations across categorical or ordinal codes could be misread as continuous clinical dose–response relationships.

**Figure 3:**
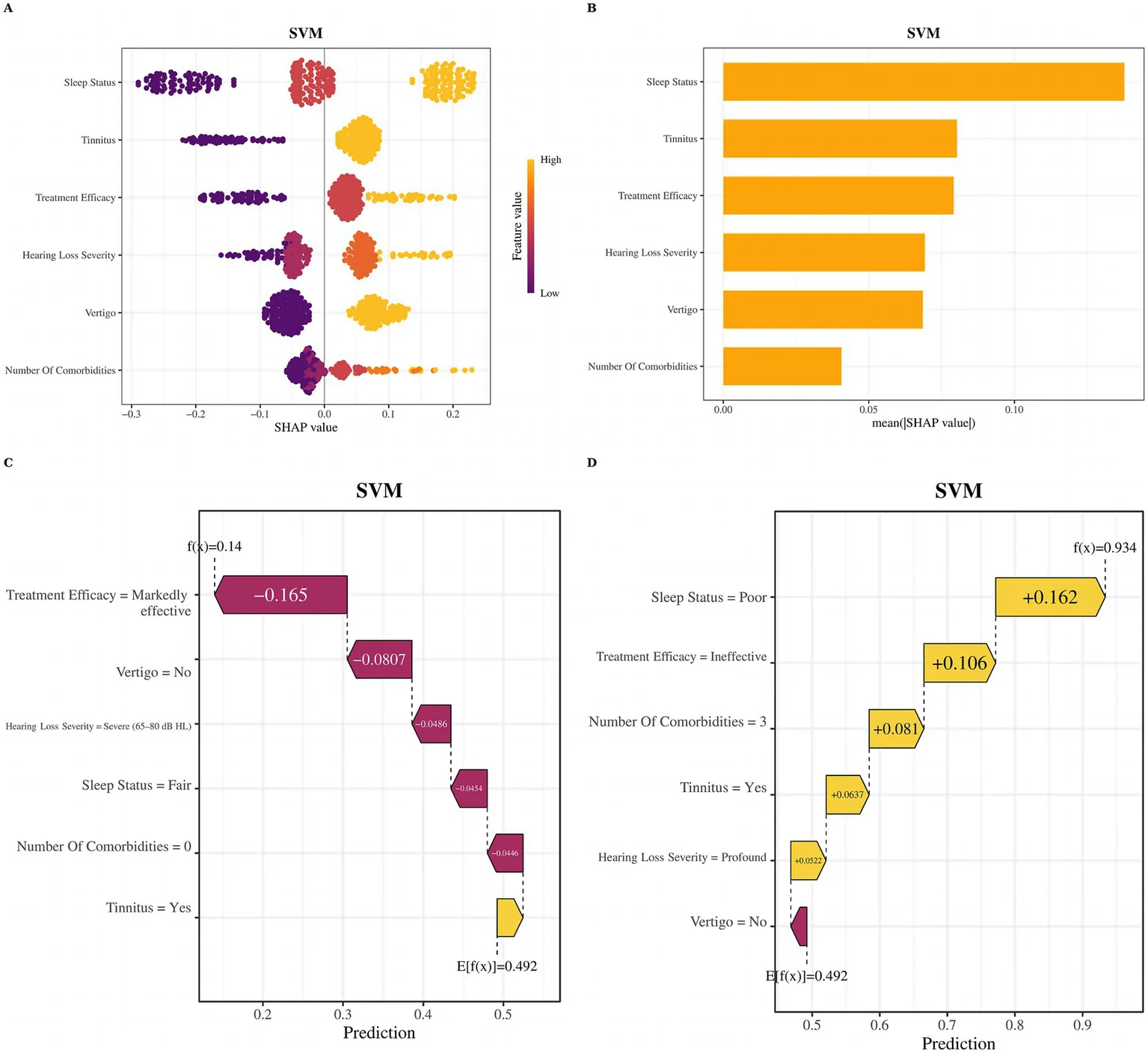
SHAP-based interpretation of the primary support vector machine model. **(A)** SHAP beeswarm plot illustrating the distribution and direction of individual feature contributions across all samples. Each point represents one observation, and color indicates the corresponding feature value from low to high. **(B)** Mean absolute SHA*p* value bar plot ranking predictors according to their overall contribution to the model output. **(C)** SHAP waterfall plot for the representative sample in row 76, showing how individual features shift the prediction from the baseline expectation to the final model output. **(D)** SHAP waterfall plot for the representative sample in row 86, showing the feature-specific positive and negative contributions to the predicted outcome. SHAP, Shapley additive explanations; SVM, support vector machine.

For the representative observation in row 76, the model output was f(x) = 0.140 relative to an expected output of E[f(x)] = 0.492. Markedly effective treatment (−0.165), absence of vertigo (−0.0807), severe hearing loss (−0.0486), fair sleep status (−0.0454), and zero comorbidities (−0.0446) shifted the output downward, whereas tinnitus contributed positively (+0.0323) (Figure 3C; Supplementary Figure S6A).

For the representative observation in row 86, the model output increased from E[f(x)] = 0.492 to f(x) = 0.934. The largest positive contributions were poor sleep status (+0.162), ineffective treatment (+0.106), three comorbidities (+0.081), tinnitus (+0.0637), and profound hearing loss (+0.0522), while absence of vertigo contributed slightly in the negative direction (Figure 3D; Supplementary Figure S6B). These plots describe model attribution for individual predictions and were not used for causal inference.

## 4 Discussion

### 4.1 Principal findings

This study developed and temporally evaluated an explainable machine-learning framework for identifying a study-specific HAM-A symptom criterion among adults hospitalized with first-episode SSNHL after initial treatment and before discharge. In the primary analysis, the six-predictor SVM achieved ROC-AUC 0.772 in the internal model-selection subset and 0.763 in same-center temporal validation, with temporal PR-AUC 0.833 and Brier score 0.215. Item-level HAM-A data allowed the principal outcome concern to be examined directly: 28/512 patients scored exactly 7, and changing the operational definition from ≥7 to ≥8 lowered overall prevalence from 62.5 to 57.0% and attenuated the *post hoc* six-predictor temporal ROC-AUC from 0.759 to 0.730, with PR-AUC falling from 0.842 to 0.786 and Brier score increasing from 0.192 to 0.209. Continuous HAM-A modeling also showed only modest temporal prediction (R^2^ = 0.112). Together with the overlap-exclusion analyses and comparable logistic benchmarks, these findings support a cautious description of the model as an investigational proxy for the acute HAM-A symptom construct, not evidence of an independently defined psychiatric disorder or of SVM superiority.

The original dataset-specific operating points yielded relatively high sensitivity but modest specificity, and the temporal threshold was optimized within the temporal data. More informative for transport is the *post hoc* development-only threshold: with 0.285 applied unchanged to the temporal cohort, sensitivity was 0.891 and specificity 0.447, 78/102 patients would be selected for further assessment, and 7/64 criterion-positive patients would be missed. This is a triage tradeoff, not a demonstrated benefit. Direct structured anxiety screening, usual clinical judgment, and model-assisted assessment were not prospectively compared, so the current evidence cannot establish that model use saves clinically meaningful workload without unacceptable missed cases.

### 4.2 Model performance in context

The choice of SVM in the primary workflow should not be interpreted as evidence that SVM was uniformly or clinically superior. Its internal model-selection ROC-AUC exceeded LR by only 0.010 and XGBoost by 0.005, whereas XGBoost had a slightly higher PR-AUC and lower Brier score. The temporal benchmarks likewise showed comparable or better point estimates for logistic alternatives. The appropriate conclusion is therefore algorithmic uncertainty rather than dominance of SVM. The SVM is retained as the model selected by the primary workflow, while simpler models remain credible comparators that require prospective head-to-head evaluation.

The similar original ROC-AUC point estimates across the two calendar periods suggest that ranking performance did not markedly deteriorate, but they do not establish statistical equivalence, calibration, or geographic transportability. Both periods came from the same department with a largely unchanged clinical environment. Temporal validation therefore provides evidence across time at one center, not evidence that the model will transport to other hospitals, regions, or healthcare systems. External-validation guidance emphasizes assessment in the population and setting of intended deployment, including both discrimination and calibration (21).

Probability-based interpretation is also more guarded after correction of the calibration display. The original temporal Brier score of 0.215 was lower than the approximately 0.234 Brier score obtained by transporting the development prevalence as a constant risk, but the improvement was modest. In the *post hoc* refit, calibration-in-the-large and slope estimates had wide confidence intervals, reflecting the small temporal sample. DCA remains exploratory because no downstream clinical action or threshold probability was prespecified. Thus, neither AUC preservation nor DCA establishes that acting on model probabilities will improve outcomes.

### 4.3 Interpretation of retained predictors and SHAP findings

Sleep status was the largest global SHAP contributor in the original model, but its interpretation is constrained by criterion overlap because HAM-A includes an insomnia item. Sleep status and the HAM-A insomnia item were recorded separately, and their overall Spearman correlation was weak (*ρ* = 0.076), arguing against literal duplicate coding. In the *post hoc* sensitivity analysis, excluding sleep status reduced temporal ROC-AUC only slightly (0.759 to 0.754), indicating that the model signal was not entirely dependent on sleep. Nevertheless, the insomnia item still contributes to the HAM-A total, so sleep-related SHAP importance should be described as contribution to the HAM-A-based classification rather than evidence that an independent sleep construct predicts psychiatric anxiety.

Tinnitus was the second-ranked SHAP contributor, and HAM-A also includes tinnitus within its sensory-symptom item. Tinnitus and the corresponding HAM-A somatic-sensory item were recorded separately, with a weak overall Spearman correlation (*ρ* = 0.070), but semantic criterion overlap remains because the HAM-A item contributes to the total score. Broader otologic literature nevertheless documents substantial psychological burden associated with tinnitus (22). The *post hoc* model that removed sleep status, tinnitus, and vertigo together retained temporal ROC-AUC 0.743, suggesting residual information from hearing-loss severity, comorbidity count, and treatment efficacy while also showing that overlapping acute symptoms contribute to discrimination.

Hearing-loss severity also contributed to model output, although its global SHAP magnitude was smaller than those of sleep status, tinnitus, and treatment efficacy. Independent evidence from an otolaryngology cohort has shown that progressively worse hearing thresholds were associated with higher anxiety and other negative emotional-state scores after adjustment for demographic characteristics (23). The population and chronicity in that study differ from those in acute SSNHL, so direct effect-size comparison is inappropriate; nevertheless, the finding supports the broader concept that greater auditory impairment can carry psychological consequences. In an acute setting, more severe hearing loss may intensify communication difficulty, uncertainty about recovery, and perceived loss of control, all of which could increase the probability that clinically relevant anxiety symptoms are detected.

Vertigo was another retained feature, and HAM-A includes giddiness within an autonomic item; these constructs are not identical but partially overlap symptomatically. Vertigo and the HAM-A autonomic item were recorded separately, with weak overall correlation (*ρ* = 0.034), which again indicates that the predictor was not a direct copy of the HAM-A item. Recent evidence in acute sensorineural hearing loss has associated greater vertigo burden with psychological distress (24). Because verbatim item-by-item adjudication wording was not retained, the relative contributions of vestibular distress and criterion contamination cannot be fully disentangled. The *post hoc* non-overlap model therefore serves as a sensitivity analysis rather than proof of construct independence.

Treatment efficacy was the third-ranked predictor by mean absolute SHAP value. In the representative explanations, markedly effective treatment shifted the model output downward, whereas ineffective treatment shifted it upward. Because treatment response was assessed before HAM-A administration, treatment efficacy was temporally available for the intended post-treatment, pre-discharge use of this model. It may capture persistent symptoms, uncertainty about recovery, or disappointment after an initial treatment course. However, treatment efficacy was not randomly assigned and SHA*p* values are conditional model attributions; neither the current observational design nor the SHAP analysis can establish that a poor treatment response causes anxiety. The inclusion of treatment efficacy also fixes the model’s intended use point after the initial treatment response has been evaluated and means that the current model should not be presented as an admission-time prediction tool.

Number of comorbidities made the smallest global contribution among the six retained predictors. More generally, SHAP improves transparency about the fitted model but does not establish independent risk effects or causal mechanisms (25). This caution is especially important here because several predictor constructs overlap with the outcome scale. Smoothed dependence plots with turning-point annotations were not retained because the underlying predictors were binary or ordinal coded and the interpolated intermediate values did not represent clinically observed states.

### 4.4 Clinical implications and model usability

If further validated, the model could fit a narrow and clinically interpretable point in the SSNHL care pathway: after the initial treatment course and treatment-response assessment, but before discharge. At that point, otolaryngology physicians or nurses could assemble the six required inputs from routine clinical information and use the model output to identify patients for whom a formal, structured psychological reassessment or mental-health consultation may be considered. A positive model output should not itself trigger a psychiatric diagnosis or treatment decision. This distinction is especially important because the HAM-A outcome used here represents an operational symptom classification and the model was not evaluated against a formal diagnostic interview for an anxiety disorder.

Implementation would also require explicit safeguards for input-data quality. The source clinical assessment forms and clinical fields were checked during routine care, and incomplete or ambiguous information was clarified at that time; consequently, the model was trained only on complete inputs and no imputation strategy was evaluated. An implementation system should therefore not silently substitute or *ad hoc* impute a missing hearing-loss category, tinnitus or vertigo status, sleep status, comorbidity count, or treatment-efficacy category. Missing or implausible inputs should instead trigger source-data verification or defer model scoring until the required information is available. Users would need basic training in the study’s predictor definitions and coding rules, particularly the categorization of hearing-loss severity, sleep status, and treatment efficacy. Automated extraction from an electronic health record could reduce manual entry burden, but the mapping of each electronic field to the model’s original definitions would itself require validation before use.

Neither the original F1-optimal thresholds of 0.196/0.216 nor the *post hoc* transported threshold of 0.285 should be adopted directly as a clinical cutoff. The first two were dataset-specific optimizations, while 0.285 was selected *post hoc* from development data after the temporal results had already been seen and was added only to demonstrate correct threshold transport. At 0.285, 24/102 temporal assessments would be spared but 7/64 patients meeting the study HAM-A criterion would be missed, with NPV 0.708. Whether that tradeoff is acceptable depends on a defined downstream action, resource constraints, and the relative consequences of missed and unnecessary assessments. Prospective comparison with usual clinical judgment and a brief direct anxiety screen is required before claiming workflow advantage.

### 4.5 Strengths and limitations

Strengths include temporally separated same-center data, comparison with logistic regression, multiple performance measures, explicit outcome and symptom-overlap sensitivity analyses, and full-pipeline nested cross-validation that repeated feature selection, tuning, and between-algorithm selection within outer resampling. The analysis distinguishes internal model selection from unbiased evaluation, documents that only one seed-24 split was generated, corrects the disease-duration definition, reports a development-only transported threshold, and corrects calibration methodology. These steps improve transparency and quantify both predictor and algorithm-selection stability, but they do not eliminate the limitations of the retrospective single-center design.

First, this was a retrospective analysis at a single tertiary hospital, and the temporal cohort was modest (*n* = 102). Same-center temporal validation does not establish geographic generalizability. The analysis relied on eligible records with completed routine clinical assessments, and no complete screening log covering all patients treated during the source periods was available. Therefore, the size of the potentially eligible patient population and reasons for records not being represented in the analytical dataset could not be quantified, and the lower number of included records in the later period could not be attributed to specific causes. This limits assessment of selection into the analytical cohort despite the completeness of the included records.

Second, complete data among the 512 analyzed records reflect the clinical documentation and contemporaneous clarification of incomplete or ambiguous information during routine care. No record was subsequently excluded from the analytical dataset because of unresolved HAM-A or predictor missingness. Nevertheless, completeness of the included records does not establish absence of selection bias in the broader clinical population, because a complete screening denominator and reason-specific counts for records not included were unavailable. Model behavior with genuinely missing inputs also remains unevaluated.

Third, outcome construct validity remains limited despite the sensitivity analyses. The study used an operational HAM-A ≥ 7 rule that differs by one point from the commonly cited ≤7 no/minimal and 8–14 mild severity convention (16). Item-level data showed that 28/512 patients scored exactly 7 and that moving to ≥8 reduced prevalence to 57.0% and attenuated temporal discrimination; therefore, the choice of boundary has a measurable effect on case prevalence and model performance. The *post hoc* Python three-selector sensitivity analysis showed that LASSO and RF-RFE membership changed across the two thresholds, whereas the final three-method intersection remained unchanged, suggesting that the most consistently selected predictor core was relatively robust to this one-point outcome shift. Sleep, tinnitus, and vertigo were recorded separately from the corresponding HAM-A items and showed only weak predictor-item correlations, but their semantic overlap with HAM-A content persists. The study used the Chinese-language 14-item HAM-A and standardized assessor training; no completed double-rating reliability dataset was available, so no cohort-specific ICC or κ is reported. Assessors were also not blinded to routine clinical predictors. *Post hoc* exclusion analyses and ≥8 sensitivity reduce uncertainty but do not eliminate criterion-contamination or diagnostic-reference-standard concerns. No independent anxiety instrument or diagnostic reference standard was collected, so criterion validity against an external anxiety construct could not be assessed in this cohort.

Fourth, key clinical variables were operationalized using pretreatment pure-tone audiometry at 250, 500, 1,000, 2000, 3,000, 4,000, and 8,000 Hz; PTA4 based on 500/1000/2000/4000-Hz air-conduction thresholds; an index-ear rule for unilateral and bilateral cases; pretreatment hearing severity; an independent seven-day pre-admission single-item sleep question; and treatment efficacy based on fixed baseline affected frequencies with cured/markedly effective/effective/ineffective thresholds and the three-level mapping used for modeling. Disease duration was defined as onset-to-treatment and categorized as <10 versus ≥10 days. Initial treatment duration and potentially symptom-modifying treatment exposures could be verified from clinical source records, including systemic corticosteroid agent, route, daily dose and treatment duration, intratympanic steroid treatment before HAM-A, psycholeptic exposure (ATC N05), antidepressant exposure (ATC N06A), and sedative/hypnotic exposure during the 24 h before HAM-A. However, these patient-level treatment-duration and medication-exposure variables were not retained in the analytical dataset used for model development, and complete cohort-level distributions were therefore unavailable for the 512 analyzed patients. Consequently, this study cannot quantify whether differences in corticosteroid regimen, intratympanic treatment, sedative/hypnotic use, or other psychotropic exposure modified HAM-A scores or confounded associations between the retained predictors and the study-specific HAM-A criterion. This limitation is particularly relevant because treatment efficacy is itself a retained post-treatment predictor and may correlate with treatment intensity, treatment duration, or rescue therapy. Future prospective validation should capture these exposures systematically and report their distributions by development/validation period and HAM-A outcome status so that treatment-related confounding and medication-related symptom modification can be examined directly.

Fifth, development/evaluation separation in the primary workflow was imperfect because the 123-patient holdout was used to choose among eight algorithms and feature selection preceded repeated model-tuning folds. Full-pipeline nested cross-validation directly addresses this source of internal optimism by recombining all 410 development observations and rerunning feature selection, preprocessing, tuning, and winner selection within each outer-training fold. Nested pooled discrimination remained moderate (ROC-AUC 0.747; PR-AUC 0.822), but algorithm identity was unstable: XGBoost won 8/10 outer evaluations and RF 2/10, while SVM won none. In contrast, four predictors entered the final intersection in every outer evaluation and two additional symptom predictors were retained in most folds. Thus, the analysis supports a relatively stable predictor signal but not the robustness of SVM as a uniquely preferred algorithm. The nested analysis remains internal resampling evidence from the 2022 development period and cannot substitute for geographically independent validation.

Sixth, the primary threshold-dependent temporal metrics were optimized within the temporal cohort and are therefore optimistic descriptions of a dataset-specific operating point. The *post hoc* development-only transported threshold addresses this procedural issue but was not prospectively prespecified. The primary calibration graphics used bin midpoints rather than actual mean predictions; corrected *post hoc* calibration has wide uncertainty. DCA also remains exploratory because no clinical action was linked prospectively to a threshold probability. Class imbalance in the primary training set was moderate (62.4% meeting versus 37.6% not meeting the HAM-A criterion), and the primary modeling pipeline used SMOTE. Because oversampling changes the effective class distribution presented during fitting, it can affect estimated probabilities and calibration even when rank discrimination is preserved. No post-SMOTE prior-probability correction or separate recalibration was used for the primary SVM; therefore, its probability calibration and Brier score should be interpreted cautiously alongside the corrected *post hoc* calibration analyses.

Seventh, reproducibility is supported by a separate versioned analysis package containing the primary analysis code, predictor/data dictionary, fixed random seeds, resampling information, model objects required for audit, and the complete nested-validation code and outputs. The 7:3 partition is documented as a single stratified split generated in R 4.4.3 using seed 24; no alternative seeds or candidate partitions were examined, and baseline *p* values were descriptive checks performed only after the split. The nested robustness analysis was executed separately in R 4.4.2. Although these materials improve auditability, the retrospective design, same-center data, and *post hoc* nature of the nested robustness analysis remain limitations.

### 4.6 Future research

Future work should preserve completed assessor-training/reliability records and patient-level treatment-duration and medication-exposure fields together with the versioned analysis files, with particular attention to corticosteroid regimen, intratympanic steroid use, sedative/hypnotic exposure, and psycholeptic/antidepressant exposure before HAM-A. Subsequent studies should prospectively validate a locked specification in geographically independent centers, compare SVM directly with simpler regression and brief direct anxiety screening, prespecify a clinically meaningful action threshold, and measure workload, missed cases, referrals, patient-centered outcomes, and resource use. A separate admission-time model would be needed if treatment efficacy is unavailable at the desired use point.

## 5 Conclusion

In the primary workflow, an explainable six-predictor SVM was selected for identifying a study-specific HAM-A symptom criterion in adults hospitalized with first-episode SSNHL after initial treatment and before discharge. Same-center temporal discrimination was moderate, but full-pipeline nested cross-validation did not reproduce SVM as the preferred algorithm: XGBoost was selected in 8/10 outer evaluations and RF in 2/10, while pooled nested ROC-AUC was 0.747 (95% CI 0.696–0.792), PR-AUC 0.822 (95% CI 0.768–0.867), and Brier score 0.208 (95% CI 0.196–0.221). Core predictor stability was stronger than algorithm stability, with hearing-loss severity, sleep status, number of comorbidities, and treatment efficacy retained in all outer-fold intersections. Item-level HAM-A data and sensitivity analyses further showed that performance depends partly on the operational symptom definition and that overlap with acute sleep/otologic symptoms cannot be ignored. The evidence therefore supports continued investigation of a structured model-assisted assessment framework, not superiority of a specific SVM, independent psychiatric diagnosis, or an implementation-ready decision rule. Geographically independent validation, prospectively locked model/threshold specifications, explicit prospective screening logs, and prospective impact evaluation are required before routine clinical use.

## Data Availability

The raw data supporting the conclusions of this article will be made available by the authors, without undue reservation.

## Supplementary material

SUPPLEMENTARY FIGURE S1 Participant flow by calendar period. The figure separately reports the February-December 2022 development period and January-August 2023 temporal-validation period. All 410 and 102 formally enrolled participants, respectively, completed the required assessments, and post-enrollment exclusions for unresolved HAM-A/predictor missingness were zero. Because individuals who were ineligible, declined, or did not proceed were not assigned study records, pre-enrollment screened denominators and reason-specific non-enrollment counts were not available and are explicitly shown as unrecorded rather than reconstructed.

SUPPLEMENTARY FIGURE S2 Analysis chronology and data separation. The diagram distinguishes the primary 287/123 development/model-selection workflow from the later post hoc robustness analyses. In the primary temporal pathway, the SVM selected using the 123-participant internal model-selection dataset was the SVM previously fitted on the 287-participant training dataset and was applied to the 102-participant temporal-validation cohort without an all-410 refit. For full-pipeline nested cross-validation, all 410 development observations were recombined; feature selection, preprocessing, inner tuning, and algorithm selection occurred within outer-training folds, while the 102-participant temporal cohort remained excluded. A separate all-410 locked full-development sensitivity analysis was applied once to the temporal cohort without temporal retuning.

SUPPLEMENTARY FIGURE S3 LASSO-selected non-zero coefficients at λ.1se and λ.min. **(A)** Non-zero coefficients at λ.1se. **(B)** Non-zero coefficients at λ.min. Abbreviation: LASSO, least absolute shrinkage and selection operator.

SUPPLEMENTARY FIGURE S4 Pairwise DeLong comparison in the internal model-selection dataset and mean cross-validated ROC-AUC in the training dataset. **(A)** Pairwise DeLong heatmap for the 123-participant internal model-selection dataset, displayed as -log_10_(P); asterisks denote nominal unadjusted *P*<0.05. **(B)** Mean ROC-AUC from repeated 10-fold cross-validation in the training dataset. Pairwise tests are exploratory and unadjusted.

SUPPLEMENTARY FIGURE S5 Confusion matrices across the training, internal model-selection, and temporal-validation datasets. **(A–H)** Training-set confusion matrices for LR, SVM, GBM, neural network, RF, XGBoost, KNN, and LightGBM. **(I–P)** Corresponding internal model-selection matrices. (Q) Temporal-validation matrix for the originally selected SVM at the dataset-specific exploratory threshold 0.216. Internal model-selection thresholds were derived from training out-of-fold predictions and transported unchanged to the 123-participant subset.

SUPPLEMENTARY FIGURE S6 Individual-level SHAP force plots for the originally selected SVM. **(A)** Row 76: f(x)=0.140 versus E[f(x)]=0.492; Tinnitus=Yes contributed +0.0323, whereas Treatment Efficacy=Markedly effective (−0.165), Vertigo=No (−0.0807), Hearing Loss Severity=Severe (65 to <80 dB HL) (−0.0486), Sleep Status=Fair (−0.0454), and Number Of Comorbidities=0 (−0.0446) shifted the prediction downward. **(B)** Row 86: f(x)=0.934; positive contributions included Hearing Loss Severity=Profound (80 to <95 dB HL), Tinnitus=Yes, Number Of Comorbidities=3, Treatment Efficacy=Ineffective, and Sleep Status=Poor. Yellow segments increase and magenta segments decrease the model output.
