# Supplementary material for: Development and temporal validation of an explainable machine-learning model for identifying anxiety symptoms in adults with sudden sensorineural hearing loss
Source: Front Public Health. 2026 Oct 23;14:2003468. doi: 10.3389/fpubh.2026.2003468 (PMC13739516; doi:10.3389/fpubh.2026.2003468)
Supplement: Supplementary file 1 [file Table_1.docx]

**Supplementary Table S1. Baseline characteristics of the training and internal model-selection datasets after the single stratified random split**

| **Characteristic** | **Overall**  **(N=410)** | **Training dataset**  **(n=287)** | Internal model-selection dataset  **(n=123)** | **Statistic** | **P value** |
| --- | --- | --- | --- | --- | --- |
| **HAM-A criterion** |  |  |  | χ²=0.00 | 0.964 |
| Does not meet | 154 (37.6) | 108 (37.6) | 46 (37.4) |  |  |
| Meets | 256 (62.4) | 179 (62.4) | 77 (62.6) |  |  |
| **Sex** |  |  |  | χ²=0.10 | 0.746 |
| Male | 215 (52.4) | 152 (53.0) | 63 (51.2) |  |  |
| Female | 195 (47.6) | 135 (47.0) | 60 (48.8) |  |  |
| **Age** |  |  |  | χ²=0.14 | 0.706 |
| <40 years | 122 (29.8) | 87 (30.3) | 35 (28.5) |  |  |
| ≥40 years | 288 (70.2) | 200 (69.7) | 88 (71.5) |  |  |
| **Education level** |  |  |  | χ²=1.63 | 0.804 |
| Primary school or illiterate | 10 (2.4) | 7 (2.4) | 3 (2.4) |  |  |
| Junior middle/technical secondary school or below | 48 (11.7) | 31 (10.8) | 17 (13.8) |  |  |
| Senior high school or junior college | 197 (48.0) | 140 (48.8) | 57 (46.3) |  |  |
| Bachelor's degree | 128 (31.2) | 92 (32.1) | 36 (29.3) |  |  |
| Master's degree or above | 27 (6.6) | 17 (5.9) | 10 (8.1) |  |  |
| **Income level** |  |  |  | χ²=2.30 | 0.813 |
| Does not meet income | 10 (2.4) | 7 (2.4) | 3 (2.4) |  |  |
| <3,000 CNY | 15 (3.7) | 13 (4.5) | 2 (1.6) |  |  |
| 3,000–5,999 CNY | 173 (42.2) | 121 (42.2) | 52 (42.3) |  |  |
| 6,000–9,999 CNY | 118 (28.8) | 81 (28.2) | 37 (30.1) |  |  |
| 10,000–20,000 CNY | 83 (20.2) | 58 (20.2) | 25 (20.3) |  |  |
| >20,000 CNY | 11 (2.7) | 7 (2.4) | 4 (3.3) |  |  |
| **Stressful life events** |  |  |  | χ²=1.59 | 0.207 |
| Does not meet | 251 (61.2) | 170 (59.2) | 81 (65.9) |  |  |
| Meets | 159 (38.8) | 117 (40.8) | 42 (34.1) |  |  |
| **Disease duration** |  |  |  | χ²=0.64 | 0.425 |
| <10 days | 93 (22.7) | 62 (21.6) | 31 (25.2) |  |  |
| ≥10 days | 317 (77.3) | 225 (78.4) | 92 (74.8) |  |  |
| **Laterality** |  |  |  | χ²=1.19 | 0.276 |
| Unilateral | 367 (89.5) | 260 (90.6) | 107 (87.0) |  |  |
| Bilateral | 43 (10.5) | 27 (9.4) | 16 (13.0) |  |  |
| **Hearing loss severity** |  |  |  | χ²=4.67 | 0.197 |
| Moderately severe (50 to <65 dB HL) | 59 (14.4) | 45 (15.7) | 14 (11.4) |  |  |
| Severe (65 to <80 dB HL) | 153 (37.3) | 98 (34.1) | 55 (44.7) |  |  |
| Profound (80 to <95 dB HL) | 160 (39.0) | 115 (40.1) | 45 (36.6) |  |  |
| Total hearing loss (≥95 dB HL) | 38 (9.3) | 29 (10.1) | 9 (7.3) |  |  |
| **Tinnitus** |  |  |  | χ²=0.22 | 0.641 |
| Does not meet | 107 (26.1) | 73 (25.4) | 34 (27.6) |  |  |
| Meets | 303 (73.9) | 214 (74.6) | 89 (72.4) |  |  |
| **Vertigo** |  |  |  | χ²=0.15 | 0.695 |
| Does not meet | 234 (57.1) | 162 (56.4) | 72 (58.5) |  |  |
| Meets | 176 (42.9) | 125 (43.6) | 51 (41.5) |  |  |
| **Aural fullness** |  |  |  | χ²=0.23 | 0.635 |
| Does not meet | 194 (47.3) | 138 (48.1) | 56 (45.5) |  |  |
| Meets | 216 (52.7) | 149 (51.9) | 67 (54.5) |  |  |
| **Sleep status** |  |  |  | χ²=0.58 | 0.747 |
| Good | 118 (28.8) | 81 (28.2) | 37 (30.1) |  |  |
| Fair | 148 (36.1) | 107 (37.3) | 41 (33.3) |  |  |
| Poor | 144 (35.1) | 99 (34.5) | 45 (36.6) |  |  |
| **Number of comorbidities, Median (IQR)** | 1 (0–2) | 1 (0–2) | 1 (0–2) | U=18011.5 | 0.729 |
| **Treatment efficacy** |  |  |  | χ²=2.87 | 0.238 |
| Markedly effective | 117 (28.5) | 82 (28.6) | 35 (28.5) |  |  |
| Effective | 228 (55.6) | 154 (53.7) | 74 (60.2) |  |  |
| Ineffective | 65 (15.9) | 51 (17.8) | 14 (11.4) |  |  |

Note. The 410-participant development cohort was divided once, in a 7:3 ratio, using stratified random sampling by the study-specific HAM-A ≥7 criterion with reproducible random seed 24. No alternative seeds or candidate splits were evaluated. Baseline comparisons were performed descriptively after the split had been fixed and did not trigger reselection or repartitioning; P>0.05 is not interpreted as evidence of equivalence. Categorical variables were compared using Pearson's chi-square test; for sparse multi-category tables, the P value was obtained by Monte Carlo fixed-margin simulation (100,000 replicates; Monte Carlo seed=20260917). Number of comorbidities is reported as median (IQR) and compared using the two-sided Mann-Whitney U test. CNY, Chinese yuan; IQR, interquartile range; dB HL, decibels hearing level. Disease duration denotes onset-to-treatment time in days.
