# Supplementary material for: Development and temporal validation of an explainable machine-learning model for identifying anxiety symptoms in adults with sudden sensorineural hearing loss
Source: Front Public Health. 2026 Oct 23;14:2003468. doi: 10.3389/fpubh.2026.2003468 (PMC13739516; doi:10.3389/fpubh.2026.2003468)
Supplement: Supplementary file 2 [file Table_2.docx]

**Supplementary Table S2. Multicollinearity diagnostics for predictors retained after intersection of three feature-selection algorithms**

| **Predictor** | **Coding / comparison** | **VIF** | **Tolerance** |
| --- | --- | --- | --- |
| Hearing loss severity | Severe (65 to <80 dB HL) vs moderately severe (50 to <65 dB HL) | 2.261 | 0.442 |
| Hearing loss severity | Profound (80 to <95 dB HL) vs moderately severe (50 to <65 dB HL) | 2.352 | 0.425 |
| Hearing loss severity | Total hearing loss (≥95 dB HL) vs moderately severe (50 to <65 dB HL) | 1.602 | 0.624 |
| Tinnitus | Yes vs no | 1.049 | 0.953 |
| Vertigo | Yes vs no | 1.032 | 0.969 |
| Sleep status | Fair vs good | 1.551 | 0.645 |
| Sleep status | Poor vs good | 1.624 | 0.616 |
| Number of comorbidities | Per 1-unit increase | 1.089 | 0.919 |
| Treatment efficacy | Effective vs markedly effective | 1.407 | 0.711 |
| Treatment efficacy | Ineffective vs markedly effective | 1.406 | 0.711 |

Note. Categorical predictors were entered using dummy coding with the following reference categories: moderately severe hearing loss (50 to <65 dB HL), no tinnitus, no vertigo, good sleep status, and markedly effective treatment. Number of comorbidities was modeled as a count variable. VIF denotes variance inflation factor; tolerance=1/VIF. The maximum condition index of the standardized predictor matrix was 3.04. As prespecified diagnostic benchmarks, VIF <5, tolerance >0.20, and condition index <10 were considered not suggestive of problematic multicollinearity. All retained predictors satisfied these criteria.
