# Supplementary material for: Development and temporal validation of an explainable machine-learning model for identifying anxiety symptoms in adults with sudden sensorineural hearing loss
Source: Front Public Health. 2026 Oct 23;14:2003468. doi: 10.3389/fpubh.2026.2003468 (PMC13739516; doi:10.3389/fpubh.2026.2003468)
Supplement: Supplementary file 3 [file Table_3.docx]

**Supplementary Table S3. Hyperparameter search spaces and selected settings for the candidate machine-learning models**

| **Model** | **Implementation** | **Hyperparameter search space / candidate values** | **Selected / final hyperparameters** |
| --- | --- | --- | --- |
| **LR** | glm | No tuning grid. Logistic regression with binomial family. | No tunable hyperparameters. |
| **SVM** | svmRadial | sigma: {0.1, 0.01, 0.001}; C: {0.1, 0.5}. | sigma = 0.1; C = 0.1. |
| **GBM** | gbm | n.trees = 100 (fixed); interaction.depth: {2, 3}; shrinkage: {0.1, 0.01}; n.minobsinnode = 5 (fixed). | n.trees = 100; interaction.depth = 3; shrinkage = 0.1; n.minobsinnode = 5. |
| **Neural network** | nnet | size: {3, 4, 5}; decay = 0.6 (fixed). Maximum iterations (maxit) = 500. | size = 5; decay = 0.6; maxit = 500. |
| **RF** | rf | mtry: {2, 3, 4, 5}. | mtry = 2. |
| **XGBoost** | xgbTree | nrounds = 200 (fixed); max_depth: {3, 4, 5}; eta: {0.1, 0.01, 0.001}; gamma = 0.5 (fixed); colsample_bytree = 0.5 (fixed); min_child_weight = 1 (fixed); subsample = 0.6 (fixed). | nrounds = 200; max_depth = 5; eta = 0.01; gamma = 0.5; colsample_bytree = 0.5; min_child_weight = 1; subsample = 0.6. |
| **KNN** | kknn | kmax: integers 3-15; distance = 1 (fixed); kernel = "optimal" (fixed). | kmax = 10; distance = 1; kernel = "optimal". |
| **LightGBM** | lightgbm | No grid search over the listed parameters. objective = binary; metric = auc; learning_rate = 0.05; num_leaves = 31; feature_fraction = 0.8; bagging_fraction = 0.8; bagging_freq = 1; min_data_in_leaf = 10; force_col_wise = TRUE. Up to 500 boosting rounds per fold with early_stopping_rounds = 30. | Final nrounds = 16; objective = binary; metric = auc; learning_rate = 0.05; num_leaves = 31; feature_fraction = 0.8; bagging_fraction = 0.8; bagging_freq = 1; min_data_in_leaf = 10; force_col_wise = TRUE; early_stopping_rounds = 30. |

Notes: Candidate hyperparameter combinations for LR, SVM, GBM, neural network, RF, XGBoost, and KNN were evaluated within the repeated 10-fold cross-validation framework, and the optimal combination was selected by cross-validated PR-AUC. Centering and scaling were applied to LR, SVM, KNN, and the neural network; tree-based models used their native predictor scales. LightGBM used the same repeated resampling partitions with training-fold-only SMOTE, AUC monitoring, up to 500 boosting rounds, and early stopping after 30 rounds without improvement; the final number of boosting rounds was 16. The final KNN specification was kmax=10, distance=1, and kernel='optimal'. Abbreviations: LR, logistic regression; SVM, support vector machine; GBM, gradient boosting machine; RF, random forest; KNN, k-nearest neighbors; PR-AUC, area under the precision-recall curve; AUC, area under the receiver operating characteristic curve.
