# Supplementary material for: Development and temporal validation of an explainable machine-learning model for identifying anxiety symptoms in adults with sudden sensorineural hearing loss
Source: Front Public Health. 2026 Oct 23;14:2003468. doi: 10.3389/fpubh.2026.2003468 (PMC13739516; doi:10.3389/fpubh.2026.2003468)
Supplement: Supplementary file 4 [file Table_4.docx]

**Supplementary Table S4. Post hoc predictor-selection sensitivity to the HAM-A outcome threshold**

| **Method** | **HAM-A ≥7** | **HAM-A ≥8** | **Threshold-related change** |
| --- | --- | --- | --- |
| LASSO | Sex; Age; Income level; Stressful life events; Disease duration; Laterality; Hearing-loss severity; Tinnitus; Vertigo; Sleep status; Number of comorbidities; Treatment efficacy | Sex; Education level; Income level; Disease duration; Hearing-loss severity; Tinnitus; Vertigo; Sleep status; Number of comorbidities; Treatment efficacy | Changed: ≥7 only: Age, Stressful life events, Laterality; ≥8 only: Education level |
| Boruta-style shadow-feature RF | Hearing-loss severity; Sleep status; Number of comorbidities; Treatment efficacy | Hearing-loss severity; Sleep status; Number of comorbidities; Treatment efficacy | Unchanged |
| RF-RFE | Sex; Age; Education level; Income level; Stressful life events; Hearing-loss severity; Tinnitus; Vertigo; Aural fullness; Sleep status; Number of comorbidities; Treatment efficacy | Age; Education level; Income level; Hearing-loss severity; Tinnitus; Vertigo; Sleep status; Number of comorbidities; Treatment efficacy | Changed: ≥7 only: Sex, Stressful life events, Aural fullness; no predictor was unique to ≥8 |
| Three-method intersection | Hearing-loss severity; Sleep status; Number of comorbidities; Treatment efficacy | Hearing-loss severity; Sleep status; Number of comorbidities; Treatment efficacy | Unchanged |

Note. This was a post hoc threshold-sensitivity analysis using all 410 development observations and identical Python procedures under HAM-A ≥7 and ≥8. LASSO used standardized predictors and 10-fold cross-validated L1 logistic regression with binomial log-loss. The Boruta-style all-relevant random-forest procedure used 50 independent shadow-feature runs with multiplicity-adjusted hit testing and a median-importance rough fix. RF-RFE used 5-fold cross-validated ROC-AUC. Python 3.13.5, scikit-learn 1.8.0, and SciPy 1.17.0 were used. The analysis evaluates stability to the one-point outcome-threshold change and does not replace the primary R-based feature-selection analysis. Best mean 5-fold RF-RFE ROC-AUC was 0.756 under ≥7 and 0.737 under ≥8.
